# Recyclable Ligand‐Free Cobalt Catalyst for Alkoxycarbonylation of Chloroacetates

**DOI:** 10.1002/cssc.202500949

**Published:** 2025-08-06

**Authors:** Wenyu Wang, Zhusong Cao, Yuya Hu, Rui Sang, Qingshan Kong, Qicai Xue, Baoxin Zhang

**Affiliations:** ^1^ Leibniz Institute for Catalysis e.V. (LIKAT Rostock) Albert‐Einstein‐Str. 29a 18059 Rostock Germany; ^2^ SHCCIG European Research and Development GmbH Hansaallee 101 40549 Düsseldorf Germany

**Keywords:** alkoxycarbonylation, alkyl chlorides, catalyst recycling, cobalt catalyst, esters

## Abstract

Direct carbonylation of the C(sp^3^)—Cl bond remains a challenging transformation in the chemical industry. The usual use of precious metals, the high cost of ligands, and the lack of recyclability of catalysts make their industrial application on a large scale impossible. A ligand‐free cobalt‐based catalyst for the alkoxycarbonylation of chloroacetates to dialkyl malonates, which are important industrial intermediates, is reported here. This catalyst uses an extremely inexpensive precursor, CoCO_3_, with a very low catalyst loading (0.5 mol%) to achieve product yields of up to 99% with nearly 100% chemoselectivity. Solvents and previously essential phase transfer agents are not needed. Most importantly, the catalyst can be easily recycled without losing its activity and selectivity. Over 95% product yield is achieved even after 8 recycles. The feasibility of this system is demonstrated through 14 examples of the alkoxycarbonylation reaction. Fourier transform infrared spectroscopy investigations identified the active cobalt species as [Co(CO)_4_]^−^. As the first recyclable cobalt‐based catalyst, this ligand‐free system has great potential for large‐scale industrial transfer.

## Graphical abstract

The first recyclable homogeneous catalyst based on ligand‐free cobalt carbonyls for alkoxycarbonylation is reported here. Using the inexpensive precursor CoCO_3_, this system is shown to yield up to 99% alkoxycarbonylation of the C(sp^3^)—Cl bond with nearly 100% selectivity. The easy recyclability and no need for phase transfer additives make it have great potential in large‐scale industrial transformations.

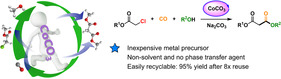

## 1 Introduction

The catalytic functionalization of organohalides is one of the most fundamental transformations in organic synthesis. It demonstrates its broad application, for example, in the Monsanto–Cativa acetic acid synthesis process^[^ ^1^ ^]^ and the Mizoroki–Heck reaction.^[^ ^2^ ^]^ The conversion occurs by oxidative addition to an organometallic compound, usually a transition metal complex.^[^ ^3^ ^]^ In most cases, the substrate is a bromide or iodide containing activated α‐carbon.^[^ ^4^, ^5^, ^6^, ^7^, ^8^, ^9^, ^10^ ^]^ Although organic chloride, especially the compound with a saturated C—Cl bond, is less expensive and more accessible to the chemical industry, its direct carbonylation is still a major challenge.^[^ ^11^ ^]^ It lacks a general protocol that is mature for large‐scale application. The reason is that neither the sp^3^‐hybrid carbon nor the hard chloride facilitates easy oxidative addition in the catalyst metal complex. Limited laboratory examples to date include alkoxycarbonylation of alkyl chlorides catalyzed by a manganese complex with PNP pincer ligands,^[^ ^12^ ^]^ carbonylation of alkyl chlorides by a bisphosphine‐rhodium complex,^[^ ^13^, ^14^ ^]^ and palladium‐catalyzed carbonylation of allylic chlorides.^[^ ^15^ ^]^ However, the use of expensive metals or ligands, high catalyst loadings, and lack of catalyst recyclability limit the feasibility of such processes for industrial applications.

**Figure 1:**
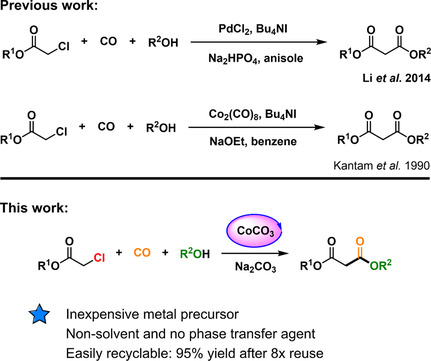
Preparation of dialkyl malonates by alkoxycarbonylation from organochlorides.

Organochlorides can be transformed into esters by catalytic alkoxycarbonylation. Synthetic esters have a wide range of applications in both bulk and fine chemical industries.^[^ ^8^ ^]^ Of these, dialkyl malonates are particularly valuable as they serve as important building blocks in the synthesis of pharmaceuticals, plastics, pesticides, fragrances, flavors, and dyes.^[^ ^16^, ^17^ ^]^ However, their industrial production typically involves the use of sodium cyanide, which is associated with cumbersome handling procedures and potential environmental contamination.^[^ ^16^, ^18^, ^19^ ^]^ Direct alkoxycarbonylation of chloroacetates using carbon monoxide, currently the most common C1 feedstock,^[^ ^20^, ^21^ ^]^ is a more environmentally friendly and straightforward alternative. However, only a few examples of this route have been reported. Li et al. recently reported the use of palladium complexes as catalysts for the production of dialkyl malonates from organic chlorides.^[^ ^22^ ^]^ Alkoxycarbonylation of chloroacetates with cobalt tetracarbonyl was reported in 1990.^[^ ^23^ ^]^ The catalyst formation requires either NaOEt for Co_2_(CO)_8_ as a precursor or NaBH_4_ for CoCl_2_. Both Pd‐ and Co‐based systems require a phase transfer reagent^[^ ^24^ ^]^ as a cocatalyst to initiate catalytic cycles (**Figure** **1**).

Our previous study of the activity and stability of unmodified cobalt carbonyls encourages us to investigate the feasibility of the inexpensive cobalt‐based catalyst for this challenging conversion.^[^ ^25^ ^]^ Herein, we report for the first time a recyclable cobalt catalyst system without ligand and phase transfer cocatalyst for the direct alkoxycarbonylation of the C(sp^3^)—Cl bond. With a very low catalyst loading (0.5 mol%), using a cheap precursor CoCO_3_ (0.38 € g^−1^, 0.83 € g^−1^ Co, Merck), this catalyst is able to achieve yields of up to 99% of dimethyl malonate (DMM) from methyl chloroacetate (MCA) with almost 100% chemoselectivity. Most excitingly, the catalyst can be easily recycled in the air. And the DMM yield remained above 95% after 8 reuses. To the best of our knowledge, this is the first recyclable, ligand‐free, non‐noble metal‐based catalyst system that provides highly selective carbonylation of the C(sp^3^)—Cl bond.

## 2 Results and Discussion

### 2.1 Optimize Reaction Conditions

At the beginning of our work, we chose the highly industrially relevant conversion of MCA to DMM by methoxycarbonylation as a model reaction for optimizing the reaction conditions (**Table** **1**). Co_2_(CO)_8_ was used as the catalyst precursor, since it can be surely converted to [Co(CO)_4_]^−^ by disproportionation in a basic environment, which is believed to be the active catalyst.^[^ ^26^ ^]^ It is noteworthy that this process can be performed in pure MeOH with a 97% yield of DMM using only 0.5 mol% of Co_2_(CO)_8_, at 90 °C and 25 bar of CO (Entry 1 Table 1, Figure S1a–c, Supporting Information). Control experiments revealed that the use of Na_2_CO_3_ as a base is essential for the success of the reaction (Entries 2−8). More soluble and stronger bases, such as K_2_CO_3_, Cs_2_CO_3_, and organic bases, result in direct formation of the by‐product methyl 2‐methoxyacetate without carbonylation. A slight excess of Na_2_CO_3_ is required to ensure complete reaction of MCA. A DMM yield of more than 95% can be obtained even when the amounts of MeOH and Co_2_(CO)_8_ are reduced to as low as 2 equiv. and 0.25 mol%, respectively (entries 9–12). Meanwhile, the CO pressure must be as high as 35 bar to ensure the gas–liquid transport balance and the stability of the Co active species in such concentrated substrates. The solvent effect was also investigated in the reaction. Yields were not significantly changed when using 1,4‐dioxane, toluene, or diethyl ether as the solvent (entries 13–14, 16). A slight decrease of yields was observed when changing solvents to THF, acetone, or acetonitrile (entries 15 and 17–18). Varying the solvent/MeOH volume ratio from 0 to 1 does not affect the DMM yield, which was over 95% when 1,4‐dioxane and toluene were tested (Figure S1d, Supporting Information).

**Table 1:** Optimization of the reaction conditions.

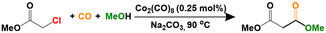					
Entrya)	Co_2_(CO)_8_ [mol%]	MeOH [equiv]	Solvent	Base	Yield [%]
1^[a]^	0.5	4	–	Na_2_CO_3_	97
2^[a]^	0.5	4	–	K_2_CO_3_	63
3^[a]^	0.5	4	–	Cs_2_CO_3_	37
4^[b]^	0.5	4	–	NaHCO_3_	44
5^[b]^	0.5	4	–	NaOH	32
6^[b]^	0.5	4	–	Triethylamine	41
7^[b]^	0.5	4	–	Pyridine	27
8^[c]^	0.5	4	–	Na_3_PO_4_	77
9^[d]^	0.5	2	–	Na_2_CO_3_	90
10^[e]^	0.5	2	–	Na_2_CO_3_	99
11^[e]^	0.25	2	–	Na_2_CO_3_	95
12^[e]^	0.1	2	–	Na_2_CO_3_	85
13^[f]^	0.25	2	1,4‐Dioxane	Na_2_CO_3_	97
14^[f]^	0.25	2	Toluene	Na_2_CO_3_	96
15^[f]^	0.25	2	THF	Na_2_CO_3_	88
16^[f]^	0.25	2	Diethyl ether	Na_2_CO_3_	96
17^[f]^	0.25	2	Acetonitrile	Na_2_CO_3_	74
18^[f]^	0.25	2	Acetone	Na_2_CO_3_	90

a) Reaction conditions: [a] MCA (11.2 mmol), MeOH (44.8 mmol), base (6.2 mmol), 90 °C, 25 bar CO, 6 h. [b] base (12.3 mmol), others same as a. [c] base (4.1 mmol), others same as a. [d] MCA (11.2 mmol), MeOH (22.4 mmol), Na_2_CO_3_ (6.2 mmol), 90 °C, 35 bar CO, 6 h. [e] MCA (11.2 mmol), MeOH (22.4 mmol), Na_2_CO_3_ (6.2 mmol), 90 °C, 35 bar CO, 9 h. [f] MCA (11.2 mmol), MeOH (22.4 mmol), Na_2_CO_3_ (6.2 mmol), Solvent (1 mL), 90 °C, 35 bar CO, 9 h.

### 2.2 Screening of Cobalt Precursors

Industrial production usually requires stable and inexpensive raw materials. The relative high cost of Co_2_(CO)_8_ compared to other cobalt salts (Table S1, Supporting Information) and its instability in air make it difficult to transfer this most used laboratory catalyst precursor to the chemical industry. Previous reports have demonstrated the preparation of cobalt carbonyl species from Co salts by reduction under H_2_/CO gas.^[^ ^27^, ^28^ ^]^ However, this method typically requires high temperatures (around 200 °C) and elevated pressures (200–300 bar). Our group's previous research reported that Co(acac)_2_ can be used to prepare Co_2_(CO)_8_ under syngas for hydroformylation of alkenes under relatively mild conditions.^[^ ^25^ ^]^ Based on this, we investigated various inexpensive and readily available Co salts as alternative catalyst precursors. Co(acac)_2_ was firstly tested. After being treated with 50 bar of syngas at 160 °C for 1 h, it was injected into the substrate mixture to catalyze the production of DMM. To our delight, DMM yields of 95% and 96% were achieved when the precursor was activated in 1,4‐dioxane and toluene, respectively (**Table** **2**, entries 1–2). These results show a catalytic performance comparable to that of Co_2_(CO)_8_, indicating a total activation to the cobalt carbonyls. Surprisingly, after pretreatment in syngas for 4 h (Figure S2, Supporting Information), CoCO_3_ can be completely reduced without any promoter in 1,4‐dioxane, while NaOH was necessary when the preformation was done in toluene (entries 3–5). Both CoCl_2_ and Co(OAc)_2_ could be easily converted to Co_2_(CO)_8_ with the appropriate bases, giving good yields in the subsequent production of DMM (entries 6–9). Notably, all precursors tested could not be activated when MeOH was used as a solvent (Table S2, Supporting Information). We decided to use CoCO_3_ as the precursor because it is the cheapest among the four Co salts. (Table S1, Supporting Information).

**Table 2:** Various cobalt precursors.

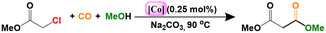		
Entrya)	Catalyst precursor	DMM yield [%]
1^[a]^	Co(acac)_2_	96
2^[b]^	Co(acac)_2_	95
3^[c]^	CoCO_3_	93
4^[d]^	CoCO_3_	32
5^[e]^	CoCO_3_ + NaOH	95
6^[c]^	CoCl_2_	–
7^[f]^	CoCl_2_ + Na_2_CO_3_	91
8^[c]^	Co(OAc)_2_	51
9^[g]^	Co(OAc)_2_ + NaOH	96

a) Catalyst preparation: [a] Co salt (0.06 mmol), Syngas (50 bar), 1,4‐dioxane (0.5 mL), 160 °C, 1 h. [b] use toluene (0.5 mL) as solvent, others same as a. [c] preparation time 4 h, others same as a. [d] use toluene (0.5 mL) as solvent, preparation time 4 h, others same as a. [e] use toluene (0.5 mL) as solvent, base (0.06 mmol), preparation time 4 h, others same as a. [f] use base (0.06 mmol), preparation time 4 h, other same as a. [g] use base (0.1 mmol), preparation time 4 h, others same as a. Carbonylation step: MCA (11.2 mmol), MeOH (22.4 mmol), Na_2_CO_3_ (6.2 mmol), were added to the above catalyst solution, 90 °C, 35 bar CO, 9 h.

### 2.3 Catalyst Recycling

**Figure 2:**
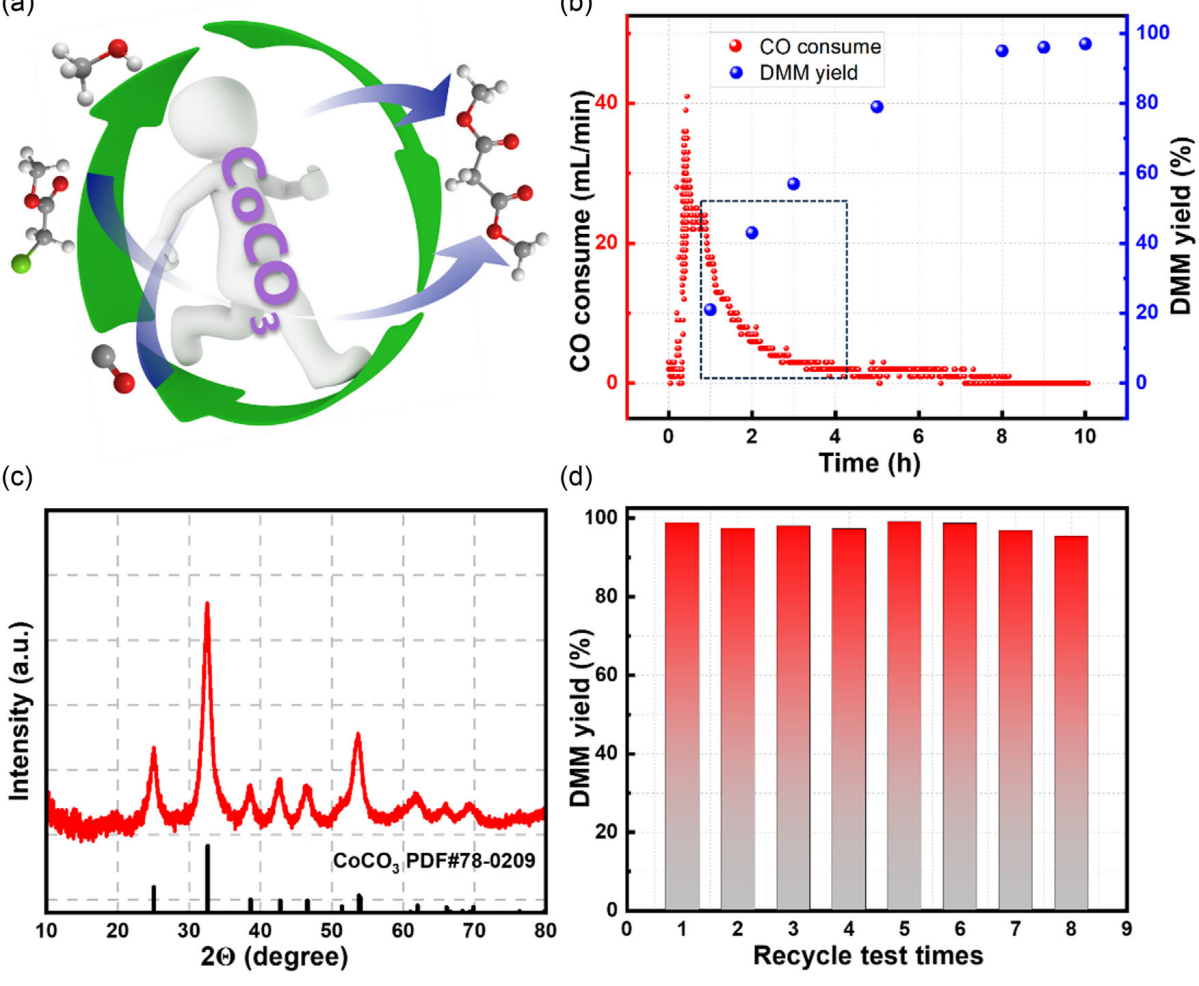
a) Reaction diagram from MCA to DMM using CoCO_3_ as catalyst precursor. b) Kinetic profile of DMM formation and CO consumption. Conditions: catalyst preparation: CoCO_3_ (0.5 mol%, 100 mg), dioxane 7.5 mL, syngas 50 bar, 160 °C, 4 h, then add MCA (170 mmol), MeOH (340 mmol), Na_2_CO_3_ (94 mmol), 35 bar CO, 0–10 h. c) The XRD pattern of recycled CoCO_3_ powder. d) Catalyst recycle test. First run condition: catalyst preparation: CoCO_3_ (1 mol%, 200 mg), dioxane 15 mL, syngas 50 bar, 160 °C, 4 h, then add MCA (170 mmol), MeOH (340 mol), Na_2_CO_3_ (98 mmol), 35 bar CO, 90 °C, 12 h.

For industrial applications, the long‐term stability and recyclability of a catalyst system are critical. Therefore, the synthesis of DMM catalyzed by CoCO_3_ on the 20‐gram scale and the recyclability of the Co catalyst were investigated (**Figure** **2**a). First, a kinetic profile of the reaction under the enhanced condition was carried out. As expected, the rates of CO consumption and DMM production were found to be consistent (Figure 2b). The CO consumption of the first 30 min was mainly contributed to the activation of Co carbonyl species, after which the reaction started. A consumption decay of CO gives an observed first order reaction rate constant (*k*_obs_) of 0.0235 min^−1^ (Figure S3a, Supporting Information). After 8 h, the substrate was completely converted and the DMM yield exceeded 95%.

A critical issue limiting the application of homogeneous catalysts is the difficulty of catalyst separation and recycling.^[^ ^29^ ^]^ There are two common industrial strategies for cobalt carbonyls: oxidation or reduction. In the bulk chemical industry, there are several patents filed in the past years describing tedious steps to recover the Co catalyst in the hydroformylation process.^[^ ^30^, ^31^, ^32^, ^33^ ^]^ However, for alkoxycarbonylation, although a number of studies on its catalysis have been published in recent years, there is no report about a recyclable catalyst developed in the laboratory that can be easily transferred to industrial application. We are very pleased to find that our CoCO_3_ system is able to meet this requirement and has great potential for large‐scale production. After the reaction, the active species, presumably [Co(CO)_4_]^−^ anion, is oxidized to Co^2+^ ions, which then precipitate as CoCO_3_ in the presence of excess Na_2_CO_3_. The organic residues are then removed by annealing at 170 °C for 4 h. Finally, water is used to wash away the inorganic salts, mainly NaCl (Figure S3b, Supporting Information), leaving pure CoCO_3_ (Figure 2c). The Co content in the sample was 48% as measured by inductively coupled plasma optical emission spectrometer (ICP‐OES). CoCO_3_ can be easily converted to Co_2_(CO)_8_ using the method described above. The cobalt retention ratio is significantly affected by the amount of Na_2_CO_3_ used in the reaction system. As the Na_2_CO_3_ usage increased from 0.55 equiv. to 0.6 equiv., the cobalt retention ratio increased from 77 to 95% (Table S3, Figure S4, Supporting Information). Most importantly, the recyclability of the CoCO_3_ catalysts was validated through an eight‐cycle recycle test using an initial CoCO_3_ concentration of 1 mol% and Na_2_CO_3_ at 0.575 equiv. (Figure 2d). During these cycles, DMM yield decreased only slightly from 98 to 95%, while cobalt retention decreased from 89 to 44% (Table S4, Supporting Information). Notably, the DMM selectivity remained at 99% throughout all eight tests, with no significant appearance of methanol substitution by‐products. These promising results demonstrate the practical feasibility of recycling and utilizing the CoCO_3_ catalyst. With optimal conditions, we next seek to explore the generality of the reaction.

### 2.4 Substrate Exploration

**Scheme 1:**
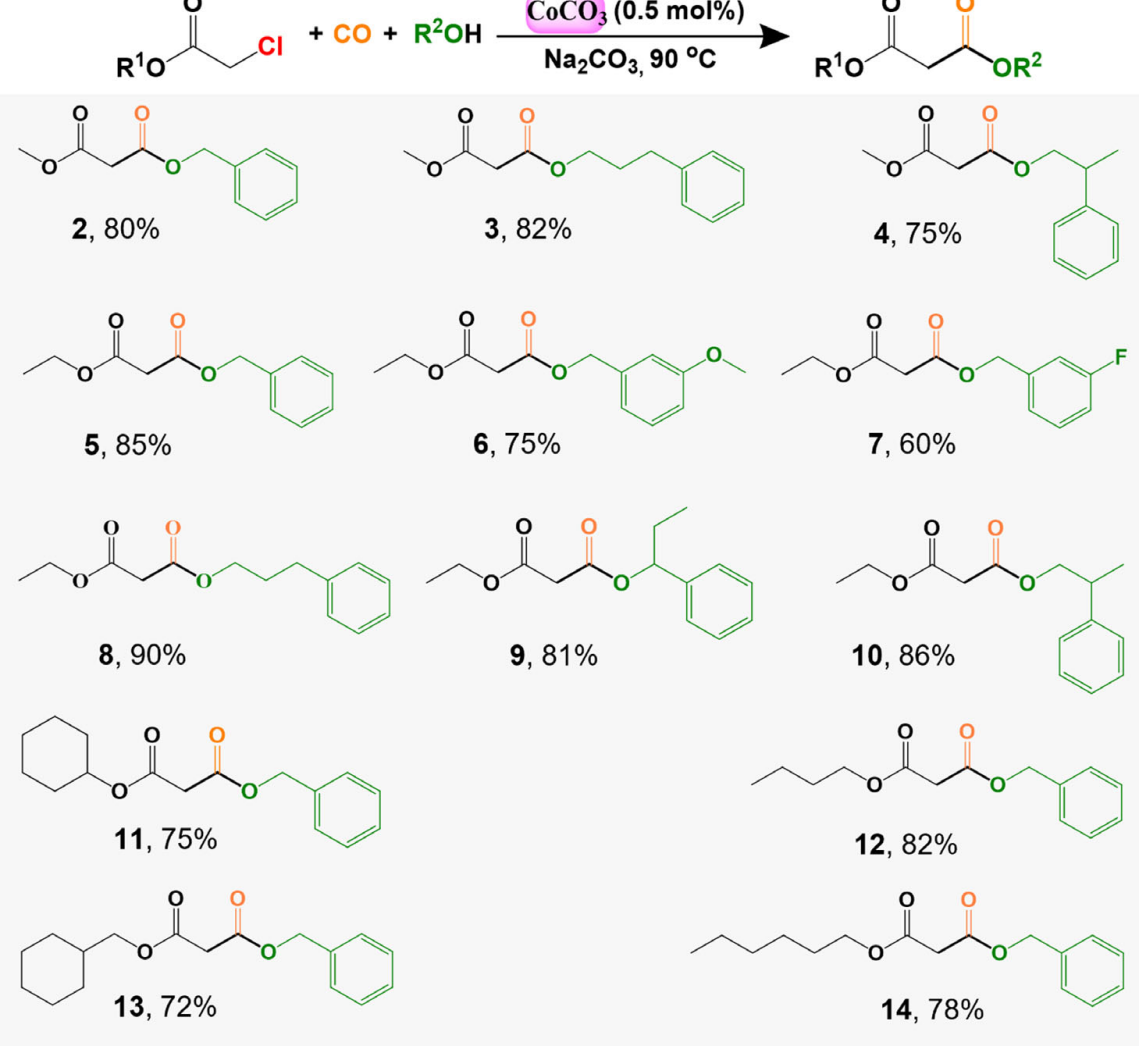
Scope of the Co‐catalyzed alkoxycarbonylation of chloroacetates with alcohols. [a] Reaction conditions: catalyst preparation: CoCO_3_ (0.02 mmol), 1,4‐dioxane (0.2 mL), 160 °C, syngas 50 bar, preparation time 4 h; after that, for reaction: chloroacetates (2.9 mmol), alcohols (5.6 mmol), Na_2_CO_3_ (1.6 mmol), were added, 90 °C, 35 bar CO, 9 h.

Under optimal conditions, we explored the generality of the reaction using a variety of alcohols, as shown in **Scheme** **1**. Our catalytic system showed great versatility, efficiently synthesizing a wide range of esters. We successfully used primary, secondary, and benzyl alcohols (**2**–**5** and **8**–**10**) as coupling partners in the carbonylation reaction. In particular, benzyl alcohols with different functional groups, such as methoxy (**6**) and fluoride (**7**), were well tolerated. In addition, we tested different types of chloroacetates, including primary and secondary alkyl chloroacetates, which participated in the reaction with good yields (**11** and **13**). The length of the carbon chain in the alkyl chloroacetates did not significantly affect the high yields obtained (**12** and **14**).

### 2.5 FTIR (Fourier Transform Infrared Spectroscopy) Investigation and Proposed Mechanism

**Figure 3:**
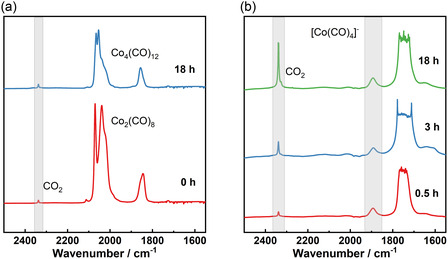
a) FTIR spectra of reaction solutions for catalyst activation using CoCO_3_ (0.1 M) with the same catalyst preparation condition of Figure 2b. The precursor was performed in 1,4‐dioxane under 50 bar of syngas at 160 °C for 4 h. The solution was measured immediately (red curve) and after storage in argon for 18 h (blue curve). b) FTIR spectra of reaction solutions of methoxycarbonylation of MCA to DMM at different reaction times. Co_2_(CO)_8_ (0.01 M) was used as the precatalyst under carbonylation conditions identical to those described in Figure 2b.

To clarify the catalytic active species and reaction mechanism, FTIR spectroscopic studies of the model reaction were conducted. Catalyst preformation starting from CoCO_3_ was carried out under 50 bar of syngas at 160 °C, and after 4 h the reaction solution was measured immediately using our FTIR spectrometer under an argon atmosphere (**Figure** **3**a, red curve). The carbonyl stretching bands of Co_2_(CO)_8_ (*ν* = 1845, 1857, 2021, 2039, 2070 cm^−1^) were observed as the only active species.^[^ ^25^, ^34^, ^35^ ^]^ This finding is consistent with our experimental results that CoCO_3_ can be converted into an active cobalt carbonyl precatalyst. The Co^2+^ is reduced by CO to Co(0) with the formation of CO_2_, whose IR band at 2337 cm^−1^ was also observed. The formed Co_2_(CO)_8_ slowly transformed under Ar to Co_4_(CO)_12_, which gives bands at 1856, 2054, and 2065 cm^−1^ (Figure 3a, blue curve). The observation of this known equilibrium further confirms the successful formation of Co_2_(CO)_8_ from CoCO_3_. Then, we measured the IR spectra of the methoxycarbonylation reaction solution of MCA to DMM under our standard conditions, using Co_2_(CO)_8_ as the precatalyst (Figure 3b). The presence of a single CO‐stretching band at 1892 cm^−1^ suggests that the active cobalt species is [Co(CO)_4_]^−1^. It is consistent with previous findings that, under basic reaction conditions as in the case of MCA to DMM, cobalt carbonyl exists in an anionic form with a cobalt oxidation state of ‐I.^[^ ^26^, ^36^ ^]^ An increased CO_2_ concentration was also observed in our measurements as the reaction progressed. This increase is attributed to the presence of CO_3_^2−^ in the system, which functions as a proton scavenger for methanol.

**Scheme 2:**
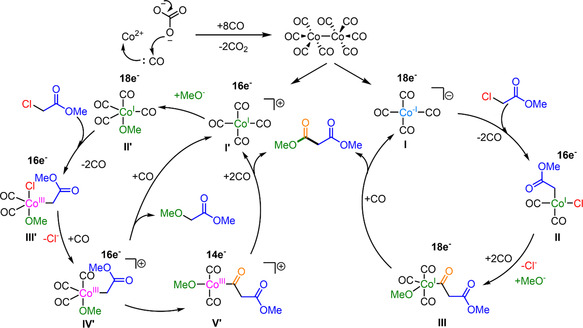
Proposed reaction mechanism.

Unlike in hydroformylation, where the cobalt catalyst is typically HCo(CO)_4_, our IR investigation confirmed that the active cobalt species is [Co(CO)_4_]^−^ under the basic reaction conditions of alkoxycarbonylation. This species is generated by the disproportionation of Co_2_(CO)_8_ formed by the reduction of CoCO_3_ by CO (**Scheme** **2**). Through oxidative addition of MCA to [Co(CO)_4_]^−^ (**II** in Scheme 2) and subsequent CO insertion and reductive elimination (**III**), the product DMM is released while the catalyst is restored (**I**). The disproportionation of Co_2_(CO)_8_, however, produces not only the Co(‐I) anion but also a 16‐electron Co(I) cation (**I’**, Scheme 2), which is immediately coordinated by a nucleophile, e.g., MeO^−^, to form a neutral 18‐electron complex (**II’**). This species can also capture the substrate by oxidative addition after CO dissociation (**III’**). Nevertheless, due to the preoccupied MeO ligand, methyl 2‐methoxyacetate as a by‐product may be formed prior to the carbonyl insertion (**IV’** and **V’**). This is in exact agreement with the observations we have made when we have used K_2_CO_3_ and Cs_2_CO_3_ as the base for the reaction (Entries 2 and 3 in Table 1). Their larger cation radii compared to Na_2_CO_3_ increases the solubility of CO_3_^2−^ in MeOH, which promotes more MeO^−^ available to form complex **II’**.

## 3 Conclusion

A new cobalt‐based catalytic system for alkoxycarbonylation of substrates with C(sp^3^)—Cl bond is presented. Using very inexpensive metal precursors, no solvents, and no phase transfer agents, the system can achieve the highest DMM yields of 99%, with nearly 100% chemoselectivity. It has been demonstrated that the catalyst is able to give a yield of more than 95% after being reused 8 times. It also shows good tolerance to both the substrate and the alcohols. Investigations using FTIR revealed that the active cobalt species is [Co(CO)_4_]^−^.To the best of our knowledge, this is the first reported recyclable catalyst system for alkoxycarbonylation based on unmodified cobalt carbonyls.

## Conflict of Interest

The authors declare no conflict of interest.

## Supporting information

Supplementary Material

## Data Availability

The data that support the findings of this study are available from the corresponding author upon reasonable request.
