# Supplementary material for: Recyclable Ligand‐Free Cobalt Catalyst for Alkoxycarbonylation of Chloroacetates
Source: ChemSusChem. 2025 Aug 6;18(17):e202500949. doi: 10.1002/cssc.202500949 (PMC12404009; doi:10.1002/cssc.202500949)
Supplement: Supplementary file 1 — Supplementary Material [file CSSC-18-e202500949-s001.pdf]

Supporting Information

Recyclable Ligand-free Cobalt catalyst for Alkoxycarbonylation of chloroacetates

Wenyu Wang,[a] Zhusong Cao,[a] Yuya Hu,[a, b] Rui Sang,*[a, b] Qingshan Kong,[b] Qicai Xue,[b] and Baoxin Zhang*[a]

[a] Dr. W. Wang, Dr. Z. Cao, Dr. Y. Hu, Dr. R. Sang, Dr. B. Zhang
Leibniz Institute for Catalysis e.V. (LIKAT Rostock)
Albert-Einstein-Str. 29a, 18059 Rostock, Germany
E-Mail: baoxin.zhang@catalysis.de
rui.sang@catalysis.de

[b] Dr. Y. Hu, Dr. R. Sang, Q. Kong, Dr. Q. Xue
SHCCIG European Research and Development GmbH
Hansaallee 101, 40549 Düsseldorf, Germany

Contents

1 General remarks 3

2. Preparation of substrates 4

3. Preparation of the catalysts 7

The preparation of $Co_2(CO)_8$ from $Co(acac)_2$ 7

The preparation of $Co_2(CO)_8$ from $Co(OAc)_2$ 7

The preparation of $Co_2(CO)_8$ from $CoCl_2$ 7

The preparation of $Co_2(CO)_8$ from $CoCO_3$ 7

4. Synthesis of DMM from MCA 8

5. Amplification and recycle experiments of MCA carbonylation 11

Amplification experiment 11

The first run 11

Recovery of catalyst 11

Recycle of reaction 11

Ex-situ FT-IR measurement 11

6. Characterization of the products 14

7. Reference 35

1 General remarks

Cobalt (II) acetate 99.99% ($Co(OAc)_2$), Cobalt (II) acetylacetonate 99.99% ($Co(acac)_2$), Cobalt (II) chloride 97% ($CoCl_2$), Methyl chloroacetate 99% (MCA), Dimethyl malonate 98% (DMM) were obtained from Sigma-Aldrich. Cobalt (II) carbonate 99.5% ($CoCO_3$), Methanol 99.8% (MeOH) anhydrous, 1,4-Dioxane 99.5% anhydrous, Dicobalt-octacarbonyl ($Co_2(CO)_8$), 95%, Sodium hydroxide-pellets (NaOH), and the alcohols, chlorides substrates were acquired from Thermo Scientific. Sodium carbonate 99.5%-100% (Na_2CO_3) was purchased from Fluka. All commercial reagents were used without further purification.

The products were characterized by ^{1}H NMR and ^{13}C NMR spectroscopy. ^{1}H and ^{13}C NMR spectra were measured on Bruker Avance 300 (300 MHz) or 400 (400M) NMR spectrometers. Chemical shifts δ (ppm) are given relative to solvent: references for $CDCl_3$ were 7.26 ppm (^{1}H-NMR) and 77.16 ppm (^{13}C-NMR). ^{13}C NMR spectra were acquired on a broad band decoupled mode. Multiples were assigned as s (singlet), d (doublet), t (triplet), q (quartet), dd (doublet of doublet), td (triplet of doublets), qd (quartet of doublets), ddt (doublet of doublet of triplets), and m (multiple).

GC analysis was performed on an Agilent HP-7890A instrument with FID detector and HP-5 capillary column. The products were measured by MS and GC analysis or isolated from the reaction mixture by solvent evaporation and further purified by column chromatography on silica gel. GC-yields were calculated using isooctane as internal standard.

Electron impact (EI) mass spectra were recorded on AMD 402 mass spectrometer (70 eV). High resolution mass spectra (HRMS) were recorded on Agilent 6210. The data are given as mass units per charge (m/z).

FTIR (Fourier Transform Infrared spectroscopy) spectra were measured on a Bruker Tensor 27 FTIR spectrometer with a liquid-nitrogen-cooled MCT-A detector. A heatable transmission flow-through IR cell (Dr. Bastian Feinwerktechnik GmbH, Wuppertal, Germany) with a CaF_2 window (Korth Kristalle GmbH, Kiel, Germany) was connected to a 25 mL Swagelok mini-reactor. Reaction solution was circulated through the IR cell and back to the autoclave by a micro gear pump (mzr-7255, HNP Mikrosysteme GmbH, Parchim, Germany). All the apparatus were connected by Swagelok quick-connects technique. OPUS v7.0 was used to control the measurement and process the acquired data. FTIR spectra ware recorded between wavenumbers of 400 and 4000 cm^{-1} with a spectral resolution of 2 cm^{-1}. Per spectrum ten scans were collected with a mirror speed set to 40 kHz.

2. Preparation of substrates

Alkyl chlorides **S1-S2** were prepared following literature procedures[1].

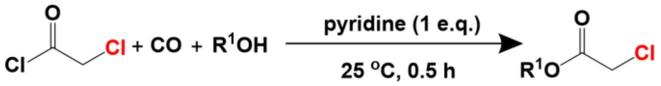

A solution of dry CH_2Cl_2 (55 mL), alcohol (43 mmol), and pyridine (3.5 mL, 43 mmol) was cooled to 0 °C with stirring, and chloroacetyl chloride (3.4 mL, 43 mmol) was added dropwise. The reaction mixture was stirred at 25 °C for 30 minutes, then quenched with cold water. The phases were separated, and the aqueous layer was extracted twice with CH_2Cl_2. The combined organic extracts were washed with water, followed by saturated salt water. The CH_2Cl_2 solution was dried over $MgSO_4$, filtered, and concentrated. The product was then purified by vacuum destillation.

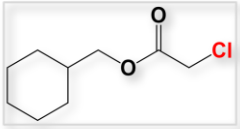

cyclohexylmethyl 2-chloroacetate (S1). Prepared according to the general procedure. Separation yield: 75 % (6.15 g), colorless oil.

^{1}H NMR (300 MHz, $CDCl_3$) δ 4.04 (s, 2H), 3.98 (d, J = 6.4 Hz, 2H), 1.74 – 1.60 (m, 6H), 1.27 – 0.91 (m, 5H).

^{13}C NMR (75 MHz, $CDCl_3$) δ 167.48, 71.31, 41.00, 37.05, 29.56, 29.54, 26.33, 25.65.

HRMS (ESI): Calcd. for $C_9H_{15}ClO_2Na^+$: 213.0653, Found: 213.0655 $[M+Na]^+$.

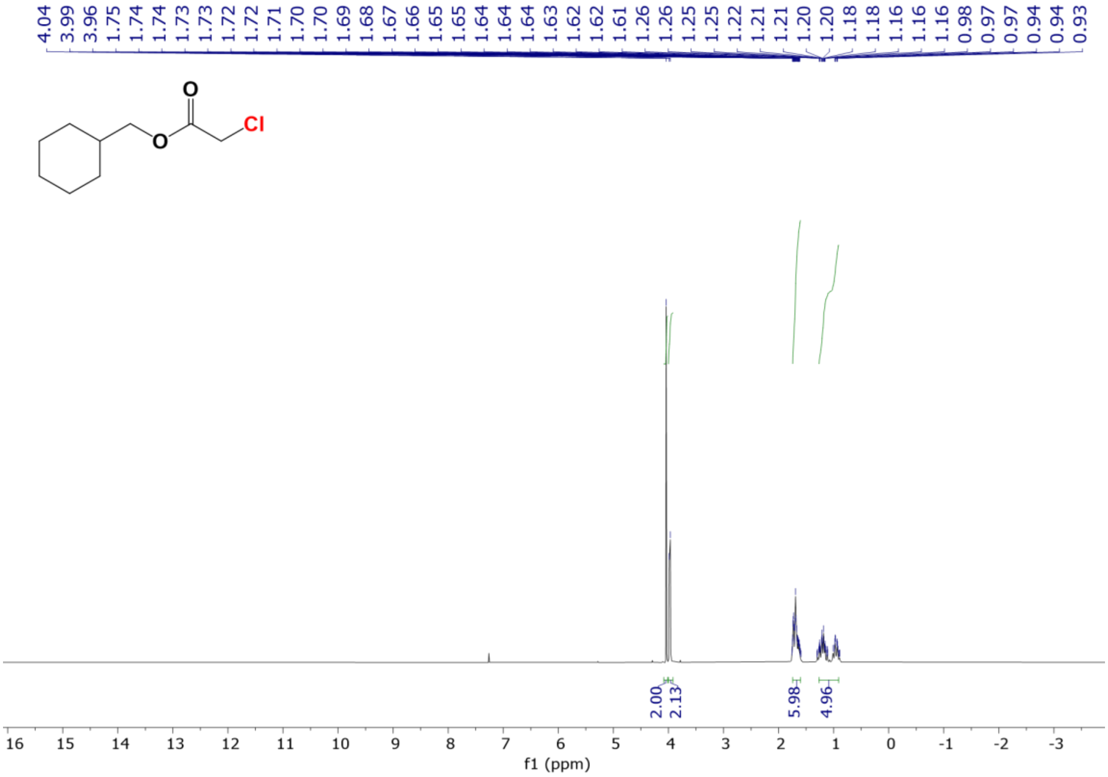

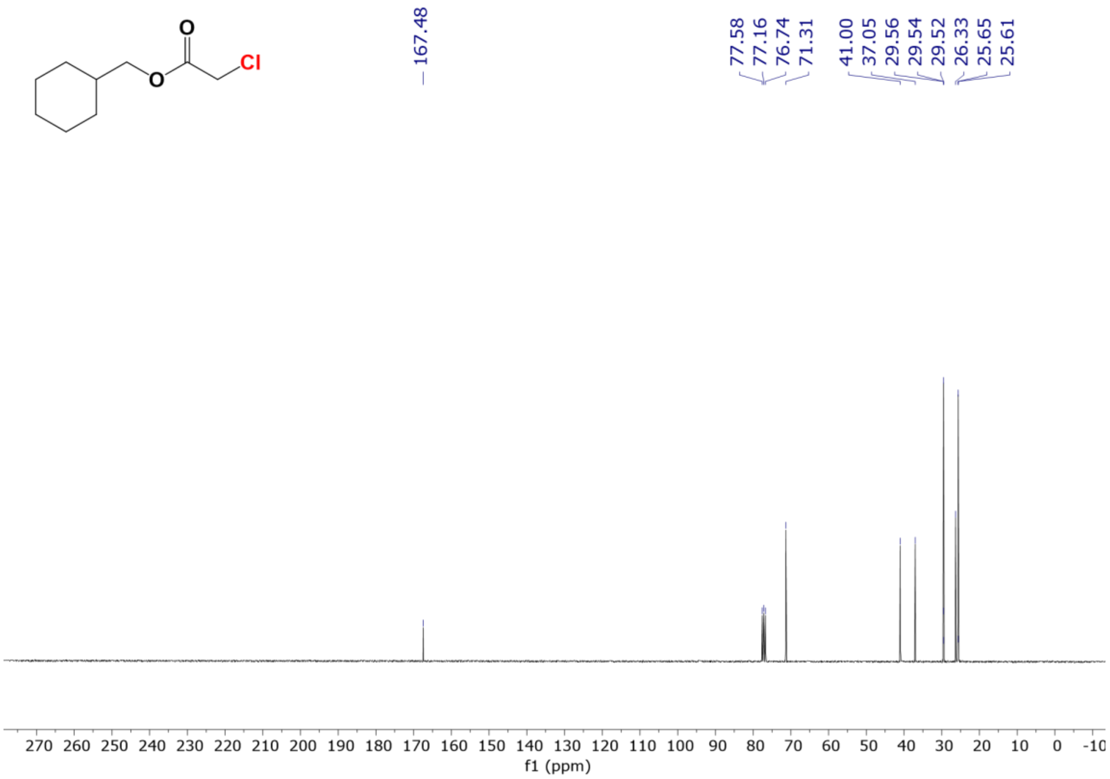

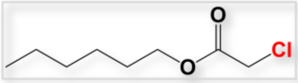

hexyl 2-chloroacetate (S2). Prepared according to the general procedure. Separation yield: 82 % (6.3 g), colorless oil.

^{1}H NMR (300 MHz, CDCl$_3$) δ 4.16 (t, J = 6.7 Hz, 2H), 4.04 (s, 2H), 1.70 – 1.59 (m, 2H), 1.39 – 1.23 (m, 6H), 0.93 – 0.82 (m, 3H).

^{13}C NMR (75 MHz, CDCl$_3$) δ 167.48, 66.49, 41.03, 31.43, 28.49, 25.51, 22.58, 14.04.

HRMS (ESI): Calcd. for $C_8H_{15}ClO_2Na^+$: 201.0653, Found: 201.0653 [M+Na]$^+$.

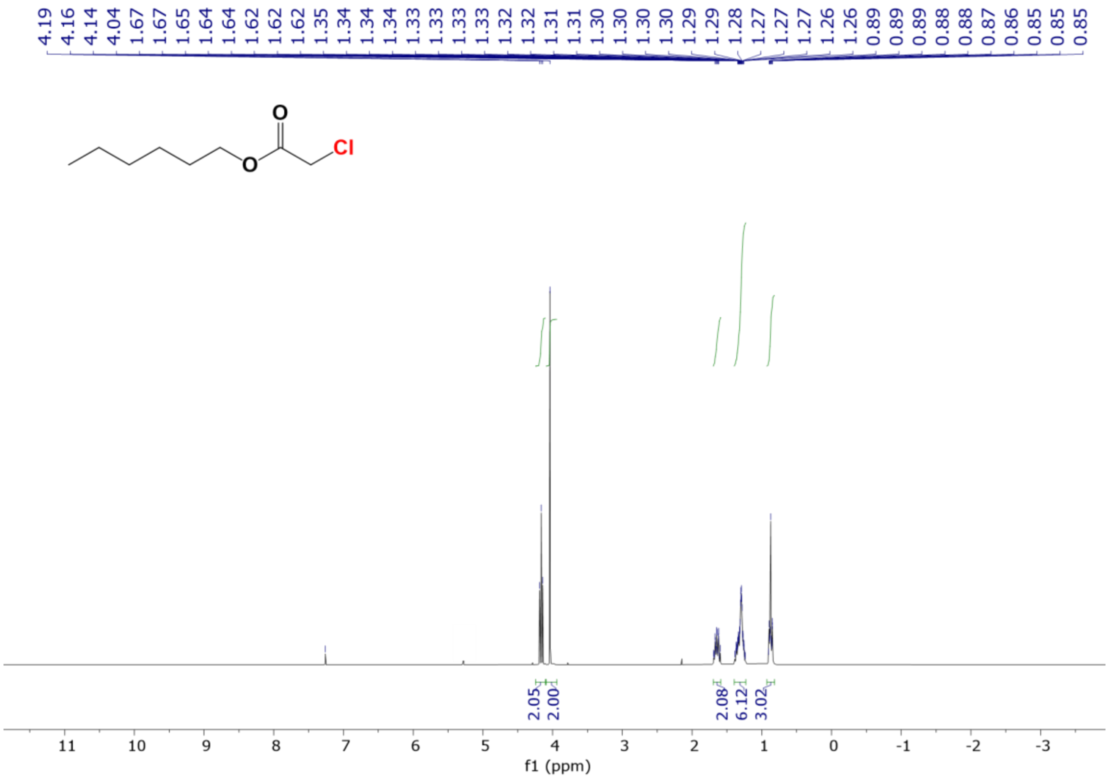
4.19
4.16
4.14
4.04
1.67
1.67
1.65
1.64
1.64
1.62
1.62
1.62
1.35
1.34
1.34
1.34
1.33
1.33
1.33
1.33
1.32
1.32
1.31
1.31
1.30
1.30
1.30
1.30
1.29
1.29
1.28
1.27
1.27
1.27
1.26
0.89
0.89
0.89
0.88
0.88
0.87
0.86
0.85
0.85
0.85
O
O
Cl
2.05
2.00
2.08
6.12
3.02
f1 (ppm)

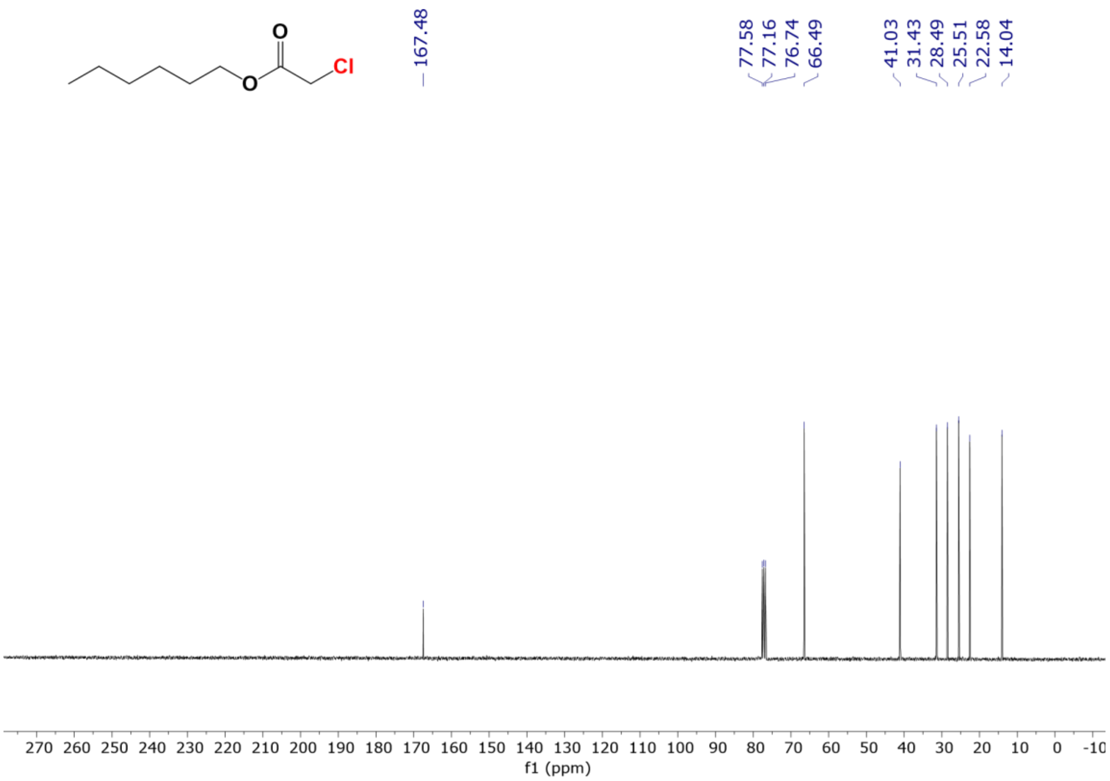
O
O
Cl
167.48
77.58
77.16
76.74
66.49
41.03
31.43
28.49
25.51
22.58
14.04
f1 (ppm)

3. Preparation of the catalysts

The preparation of $Co_2(CO)_8$ from $Co(acac)_2$

In general, cobalt acetylacetonate (0.06 mmol, 14 mg), 0.5 mL dioxane or toluene was added to 15 mL stainless steel autoclave under magnetic stirring. Then, it was filled with nitrogen (15 bar) four times, synthesis gas (15 bar) two times. After that, the reactor was pressurized synthesis gas to 35 bar, heated to 160 °C. And then the pressure was up to 50 bar. The reactor was stirred (600 rpm) for 1 h under this condition (160 °C, 50 bar synthesis gas). After the reaction was completed, the autoclave was cooled down to room temperature, the pressure was released and purged six times with 15 bar nitrogen.

The preparation of $Co_2(CO)_8$ from $Co(OAc)_2$

In general, cobalt acetate (0.06 mmol, 10 mg), NaOH (0.1 mmol, 5 mg), 0.5 mL dioxane was added to 15 mL stainless steel autoclave under magnetic stirring. Then, it was filled with nitrogen (15 bar) four times, synthesis gas (15 bar) two times. After that, the reactor was pressurized synthesis gas to 35 bar, heated to 160 °C. And then the pressure was up to 50 bar. The reactor was stirred (600 rpm) for 4 h under this condition (160 °C, 50 bar synthesis gas). After the reaction was completed, the autoclave was cooled down to room temperature, the pressure was released and purged six times with 15 bar nitrogen.

The preparation of $Co_2(CO)_8$ from $CoCl_2$

In general, cobalt chloride (0.06 mmol, 7.5 mg), Na_2CO_3 (0.06 mmol, 6 mg), 0.5 mL dioxane was added to 15 mL stainless steel autoclave under magnetic stirring. Then, it was filled with nitrogen (15 bar) four times, synthesis gas (15 bar) two times. After that, the reactor was pressurized synthesis gas to 35 bar, heated to 160 °C. And then the pressure was up to 50 bar. The reactor was stirred (600 rpm) for certain time from 4 h under this condition (160 °C, 50 bar synthesis gas). After the reaction was completed, the autoclave was cooled down to room temperature, the pressure was released and purged six times with 15 bar nitrogen.

The preparation of $Co_2(CO)_8$ from $CoCO_3$

Typically, cobalt carbonate (0.06 mmol, 6.6 mg), 0.5 mL dioxane or Toluene with NaOH (0.06 mmol, 2.3 mg) was added to 15 mL stainless steel autoclave under magnetic stirring. Then, it was filled with nitrogen (15 bar) four times, synthesis gas (15 bar) two times. After that, the reactor was pressurized synthesis gas to 35 bar, heated to 160 °C. And then the pressure was up to 50 bar. The reactor was stirred (600 rpm) for 4 h under this condition (160 °C, 50 bar synthesis gas). After the reaction was complete, the autoclave was cooled down to room temperature, the pressure was released and purged six times with 15 bar nitrogen.

4. Synthesis of DMM from MCA

The above $Co_2(CO)_8$ solution in dioxane or toluene or commercial $Co_2(CO)_8$ (0.03 mmol), methyl chloroacetate (11.2 mmol, 1 mL), MeOH (22.4 mmol, 1 mL), Na_2CO_3 (6.3 mmol, 660 mg) was added to 15 mL stainless steel autoclave. After flush four times with nitrogen (15 bar), CO (15 bar) three times, a pressure of 15 bar of CO was adjusted. Then, the temperature was up to 90 ^oC, pressure 35 bar, followed by stirring (600 rpm) for 9 h to complete this reaction. After the reaction was complete, the autoclave was cooled down to room temperature, the pressure was released and purged six times with 15 bar nitrogen.

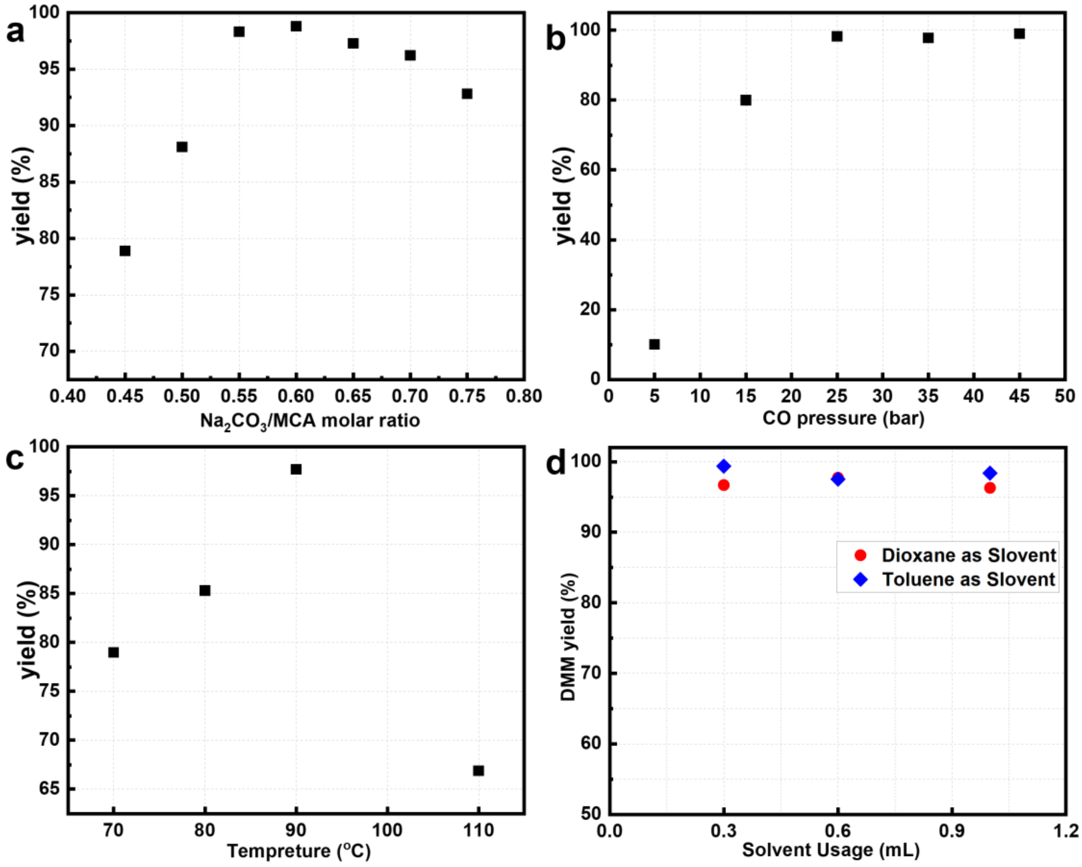

Figure S1a: Effect of Na_2CO_3/MCA molar ratio on DMM yield. MCA 11.2 mmol, MeOH 44.8 mmol as substrate, $Co_2(CO)_8$ 0.5 mol%, Na_2CO_3 5.0-8.4 mmol, CO 25 bar, 6 h. S**1b:** Effect of CO pressure on DMM yield. MCA 11.2 mmol, MeOH 44.8 mmol as substrate, $Co_2(CO)_8$ 0.5 mol%, Na_2CO_3 6.2 mmol, CO 5-45 bar, 6 h. S**1c:** The catalytic performance with different temperature. MCA11.2 mmol, MeOH 44.8 mmol as substrate, $Co_2(CO)_8$ 0.5 mol%, Na_2CO_3 6.2 mmol, CO 25 bar, 6 h. **S1d**: The catalytic performance with different solvent usage. MCA 11.2 mmol, 1 mL, MeOH 22.4 mmol as substrate, $Co_2(CO)_8$ 0.25 mol%, Na_2CO_3 6.2 mmol, 90 °C, CO 35 bar, 9 h, certain amount of Dioxane or Toluene as solvent.

Table S1: The price of different Co salts.

Salt (Co content wt%)	Price €/g	Price €/g_{Co}
$CoCO_3$ (45%)	0.38	0.83
$CoCl_2$ (43.7%)	1.08	2.47
$Co(OAc)_2$ (32.6%)	0.952	2.92
$Co(acac)_2$ (22.3%)	1.436	6.44
$Co_2(CO)_8$ (32.8%)	6.47	19.7

Price from Merck.

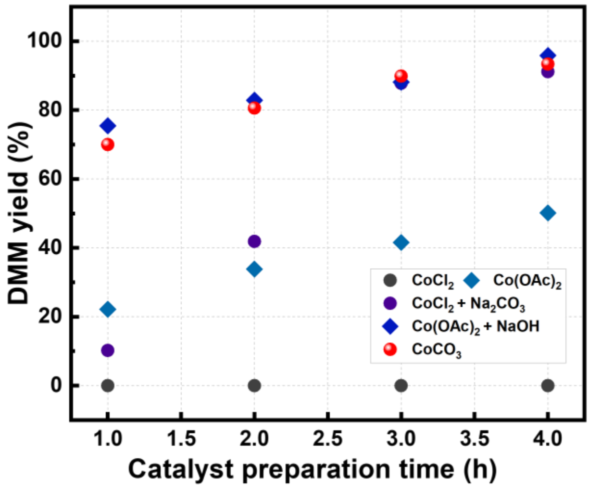

Figure S2 Effect of catalyst preparation time on DMM yield. Catalyst preparation: Co salt (0.06 mmol), Syngas (50 bar), 1,4-dioxane (0.5 mL), 160 °C, 4 h. Carbonylation step: add MCA (11.2 mmol), MeOH (22.4 mmol), Na_2CO_3 (6.2 mmol), 90 °C, 35 bar CO, 9 h.

Table S2: the preparation of cobalt carbonyls using MeOH as solvent.

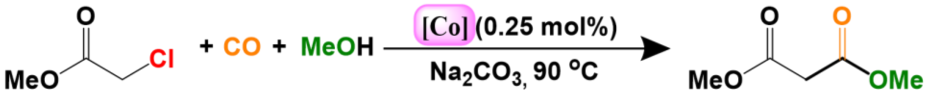

Entry	Catalyst Precursor	DMM Yield (%)
1[a]	$Co(acac)_2$	-
3[b]	$CoCO_3$	-
5[c]	$CoCO_3$+NaOH	-
6[b]	$CoCl_2$	-
7[c]	$CoCl_2$+Na_2CO_3	-
8[b]	$Co(OAc)_2$	-
9[d]	$Co(OAc)_2$+NaOH	-

Catalyst Preparation: [a] Co salt: 0.06 mmol, Syngas 50 bar, Solvent: Dioxane 0.5 mL, 160 °C, 1 h. [b] preparation time 4 h, other same as a. [c] base 0.06 mmol, preparation time 4 h, other same as a. [d] use base 0.1 mmol, preparation time 4 h, other same as a.

Carbonylation Step: all with same condition, add MCA 11.2 mmol, MeOH 11.2 mmol, Na_2CO_3 6.2 mmol, 90 °C, 35 bar CO, 9 h.

5. Amplification and recycle experiments of MCA carbonylation

Amplification experiment

In general, $Co_2(CO)_8$ in 7.5 mL dioxane or toluene made from $CoCO_3$ (0.9 mmol, 100 mg), MCA (170 mmol, 15 mL), MeOH (340 mmol, 14.0 mL), Na_2CO_3 (93.5 mmol, 9.9 g) was added to 175 mL stainless steel autoclave. After flush four times with nitrogen (15 bar), CO (15 bar) three times, a pressure of 20 bar CO was adjusted. Then, the temperature was up to 90 ^oC, pressure 35 bar, followed by stirring (600 rpm) for 9 h to complete this reaction. After the reaction was complete, the autoclave was cooled down to room temperature, the pressure was released and purged six times with 15 bar nitrogen.

The first run

$Co_2(CO)_8$ in 15 mL dioxane made from $CoCO_3$ (1.8 mmol, 200 mg), MCA (170 mmol, 15 mL), MeOH (340 mmol, 14.0 mL), Na_2CO_3 (93.5 mmol, 9.9 g) was added to 175 mL stainless steel autoclave. After flush four times with nitrogen (15 bar), CO (15 bar) three times, a pressure of 20 bar CO was adjusted. Then, the temperature was up to 90 ^oC, pressure 35 bar, followed by stirring (600 rpm) for 9 h to complete this reaction. After the reaction was complete, the autoclave was cooled down to room temperature, the pressure was released and purged six times with 15 bar nitrogen.

Recovery of catalyst

After the reaction system was purged with $N_{2,}$ add 5 bar air, 15 bar N_2 to the reaction system to oxidize of cobalt species. Then, the temperature was up to 60 ^oC, followed by stirring (600 rpm) for 6 h, precipitating the Co species as much as possible. Finally, the autoclave was cooled down to room temperature, pressure was released, and reactor was opened directly.

Next, the solid was obtained by filtered, washed with MeOH 3*5 mL for three times. Inductively coupled plasma optical emission spectroscopy (ICP-OES) indicates that Na, Co contents in this solid are 0.85%, 40.6 wt %, respectively. Then, to remove the organic residue and sinter $CoCO_3$, the solid mixture was annealed in air at 170 ^oC for 4 h. After that, the solid mixture was transferred to a 100 mL conical flask, 30 mL water was added, followed by stirring for 1 h. Dry $CoCO_3$ solid was got by filtered, washed three times with 3*10 mL of deionized water and then dried at 60 °C in vacuum for 3 h. Na, Co content in this dry $CoCO_3$ is 0.55 wt %, 45.3%, respectively, determined by ICP-OES results.

Above dry $CoCO_3$, 15 mL dioxane was added to 175 mL stainless steel autoclave. Then, it was filled with nitrogen (15 bar) four times, synthesis gas (15 bar) two times. After that, the reactor was pressurized synthesis gas to 35 bar, heated to 160 ^oC. And then the pressure was up to 50 bar. The reactor was stirred (600 rpm) for 4 h under this condition (160 ^oC, 50 bar synthesis gas). After the reaction was completed, the autoclave was cooled down to room temperature, the pressure was released and purged six times with 15 bar nitrogen.

Recycle of reaction

Then, same as the first run, the recover $Co_2(CO)_8$ cat in 15 mL dioxane, MCA (170 mol, 15 mL), MeOH (340 mol, 14 mL), Na_2CO_3 (93.5 mmol, 9.9 g) was added to 175 mL stainless steel autoclave. After flush four times with nitrogen (15 bar), CO (15 bar) three times, a pressure of 20 bar CO was adjusted. Then, the temperature was up to 90 ^oC, pressure 35 bar, followed by stirring (600 rpm) for 9 h to complete this reaction. After the reaction was complete, the autoclave was cooled down to room temperature, the pressure was released and purged six times with 15 bar nitrogen.

Ex-situ FT-IR measurement

For the measurement of catalyst: The catalyst activation was carried out under the following conditions: 50 bar syngas, 160°C, for 4 hours. Fresh $Co_2(CO)_8$ was prepared by dissolving 0.9 mmol (100 mg) of $CoCO_3$ in 7.5 mL of dioxane. The resulting solution was then injected into the 25 mL mini-reactor of the FTIR system under an argon atmosphere. Spectra were subsequently recorded.

For the measurement of reactive species: A mixture of $Co_2(CO)_8$ in 7.5 mL dioxane (prepared from the activation of $CoCO_3$ (0.9 mmol, 100 mg)), MCA (170 mmol, 15 mL), MeOH (340 mmol, 14.0 mL), and Na_2CO_3 (93.5 mmol, 9.9 g) was added to a 175 mL stainless steel autoclave. The reaction was carried out at 90°C and 35 bar CO for a specific duration, followed by cooling.

After the reaction, 10 mL of the solution was extracted under 1 bar CO and immediately injected into the 25 mL mini-reactor of the FTIR system under an argon atmosphere for FTIR spectroscopy measurement.

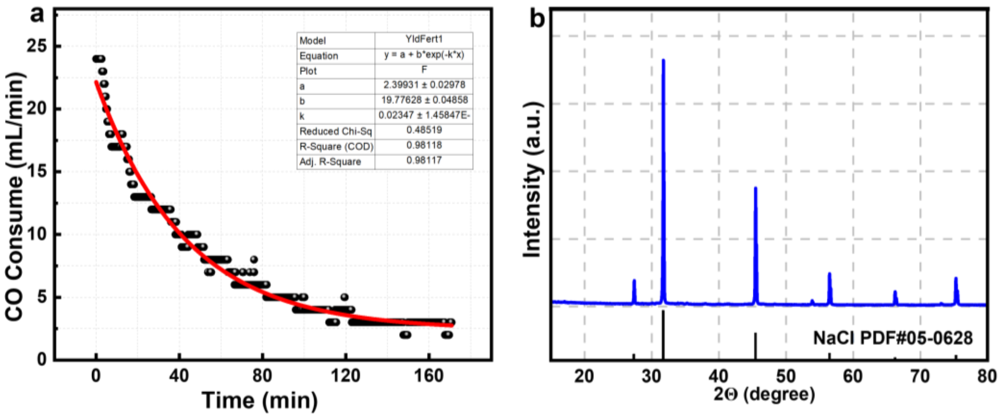

Figure S3a the exponential regression of the consumption curve of the black rectangle area of Figure 1b. **S3b** the XRD pattern of the precipitation before water wash.

Table S3: Co retention ratio with different post-treatment procedures.

Entry	Na_2CO_3 usage (equiv)	Post treatment	DMM yield (%)	Co retention ratio (%)
1	0.55	/	98	62
2	0.55	a	97	77
3	0.575	a	96	90
4	0.6	a	97	95

Condition: Catalyst preparation: $CoCO_3$ (1 mol%, 200 mg), Dioxane 15 mL, Syngas 50 bar, 160 °C, 4 h, then add MCA (170 mmol), MeOH (340 mol), Na_2CO_3 (0.55-0.6 equiv), 35 bar CO, 90°C, 12 h.[a]5 bar air, 15 bar N_2, 60 °C, 6 h.

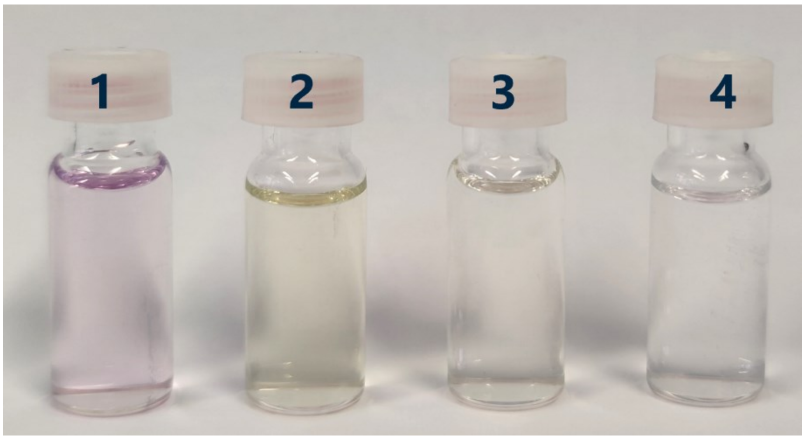

Figure S4 The real image of the reaction system after the precipatation of $CoCO_3$. (The number 1-4 corresponding to the number in Table S3.)

Table S4: Catalytic performance and Co retention ratio after each recycle run.

Entry	DMM yield (%)	DMM selectivity (%)	Co retention ratio (%)
1	98	99	89
2	97	99	80
3	98	99	72
4	97	99	65
5	98	99	60
6	98	99	54
7	97	99	49
8	95	99	44

6. Characterization of the products

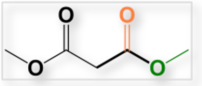

dimethyl malonate (1). Known structure[2]. Prepared according to the general procedure. The crude product was purified by silica gel chromatography (PE/EA = 5:1) to afford the title compound as a colorless oil (1.3 g, 90% yield).

^{1}H NMR (300 MHz, $CDCl_3$) δ 3.61 (s, 6H), 3.26 (m, 2H).

^{13}C NMR (75 MHz, $CDCl_3$) δ 166.75, 52.23, 40.83.

HRMS (ESI): Calcd. for $C_5H_8O_4Na^+$: 155.0315, Found: 155.0316 $[M+Na]^+$.

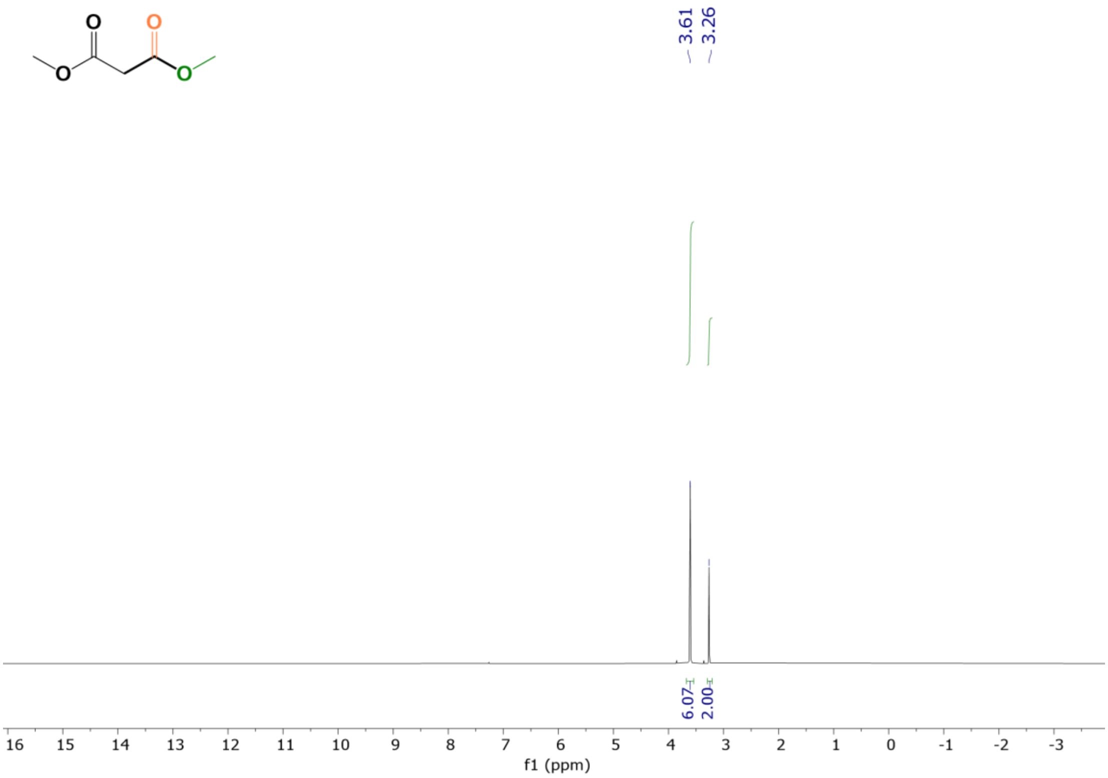

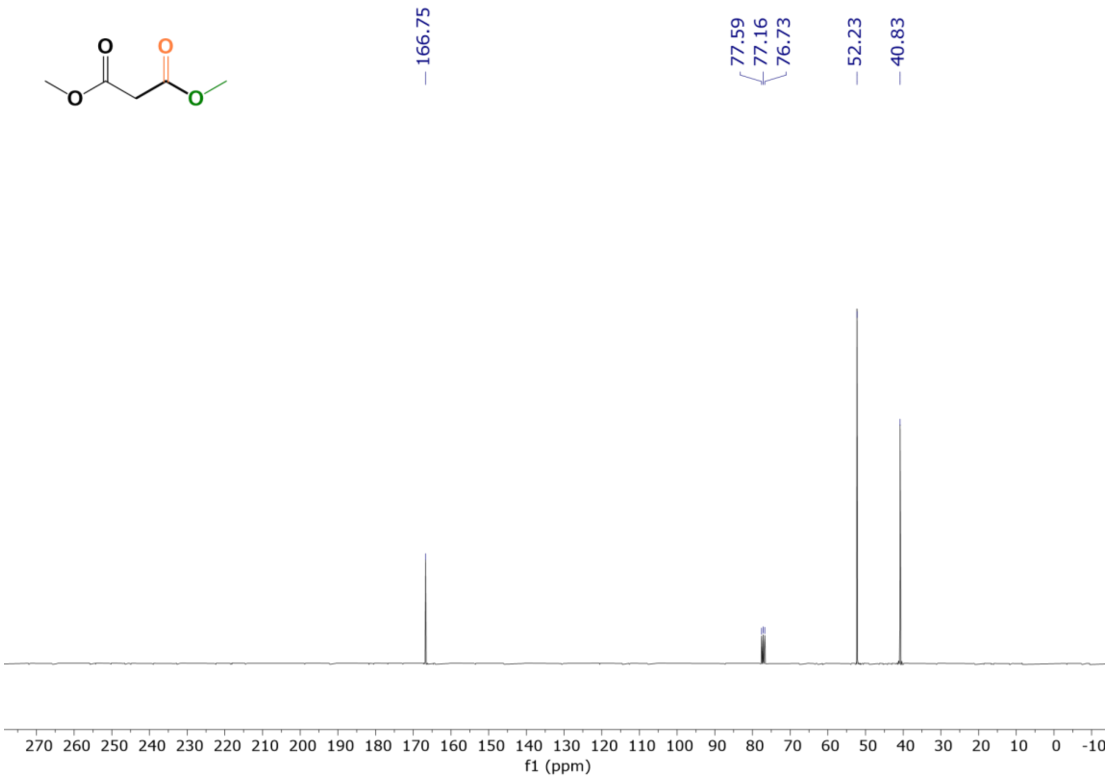

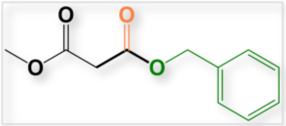

benzyl methyl malonate (2). Known structure[3]. Prepared according to the general procedure. The crude product was purified by silica gel chromatography (PE/EA = 5:1) to afford the title compound as a colorless oil (474 mg, 80% yield).

^{1}H NMR (300 MHz, $CDCl_3$) δ 7.34-7.38 (m, 5H), 5.19 (s, 2H), 3.73 (s, 3H), 3.43 (s, 2H).

^{13}C NMR (75 MHz, $CDCl_3$) δ 166.92, 166.40, 135.32, 128.66, 128.51, 128.35, 67.30, 52.58, 41.38.

HRMS (ESI): Calcd. for $C_{11}H_{12}O_4Na^+$: 231.0628, Found: 231.0628 $[M+Na]^+$.

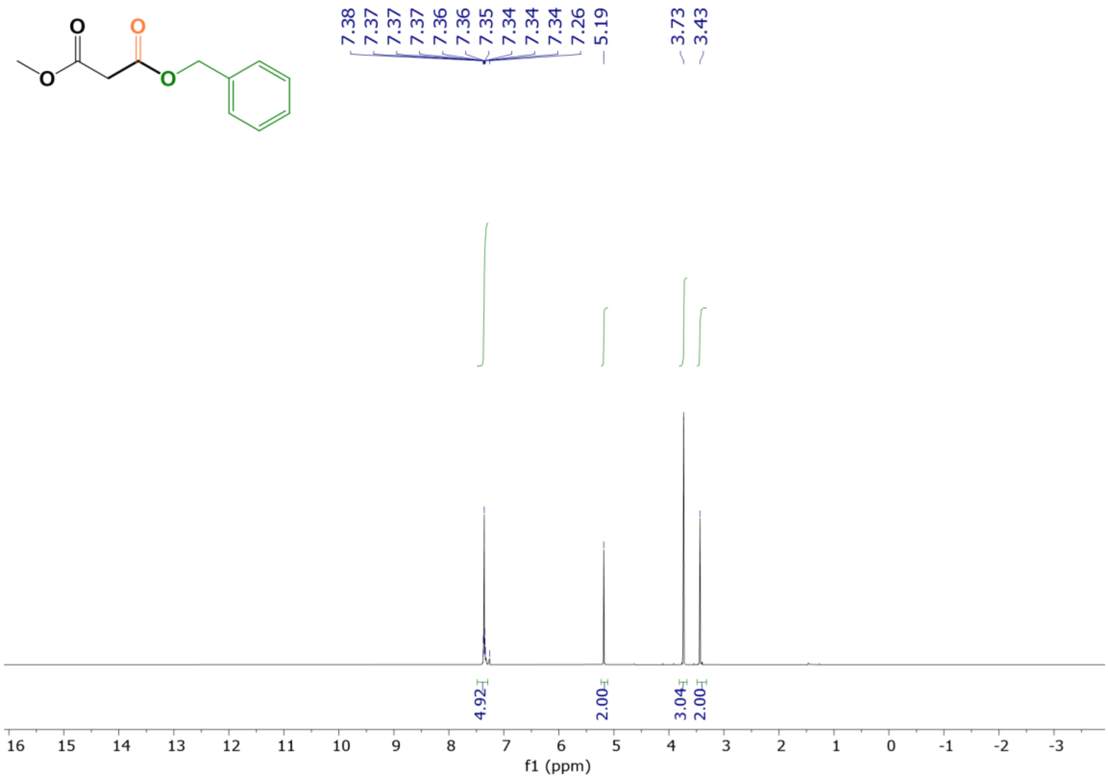
O
O
O
O
7.38
7.37
7.37
7.37
7.36
7.36
7.35
7.34
7.34
7.34
7.26
5.19
3.73
3.43
4.92
2.00
3.04
2.00
16 15 14 13 12 11 10 9 8 7 6 5 4 3 2 1 0 -1 -2 -3
f1 (ppm)

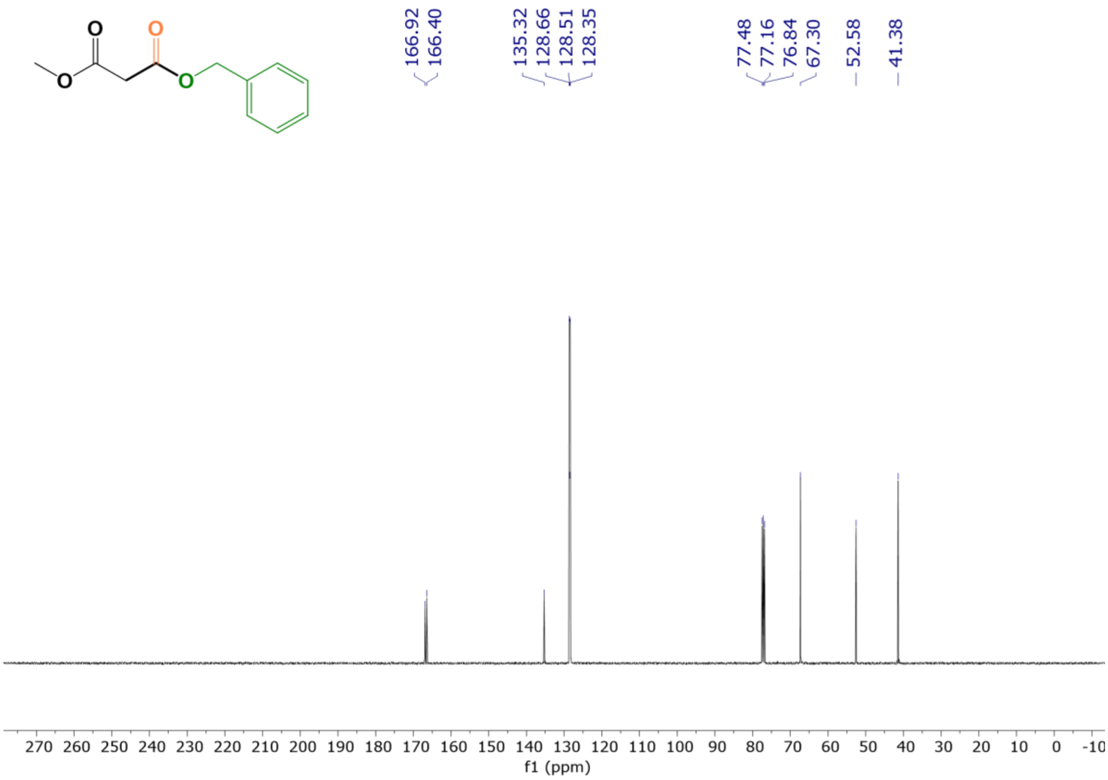
O
O
O
O
166.92
166.40
135.32
128.66
128.51
128.35
77.48
77.16
76.84
67.30
52.58
41.38
270 260 250 240 230 220 210 200 190 180 170 160 150 140 130 120 110 100 90 80 70 60 50 40 30 20 10 0 -10
f1 (ppm)

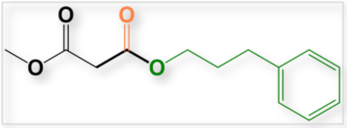

methyl (3-phenylpropyl) malonate (3). Prepared according to the general procedure. The crude product was purified by silica gel chromatography (PE/EA = 5:1) to afford the title compound as a colorless oil (552 mg, 82% yield).

^{1}H NMR (300 MHz, $CDCl_3$) δ 7.56 – 6.97 (m, 5H), 4.20 (t, *J* = 6.5 Hz, 2H), 3.78 (s, 3H), 3.42 (s, 2H), 2.84 – 2.58 (m, 2H), 2.07 – 1.94 (m, 2H).

^{13}C NMR (75 MHz, $CDCl_3$) δ 166.06, 165.55, 140.05, 127.52, 127.47, 125.13, 63.87, 51.59, 40.41, 31.06, 29.09.

HRMS (ESI): Calcd. for $C_{13}H_{16}O_4Na^+$: 259.0941, Found: 259.0941 $[M+Na]^+$.

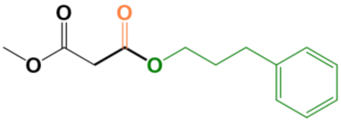

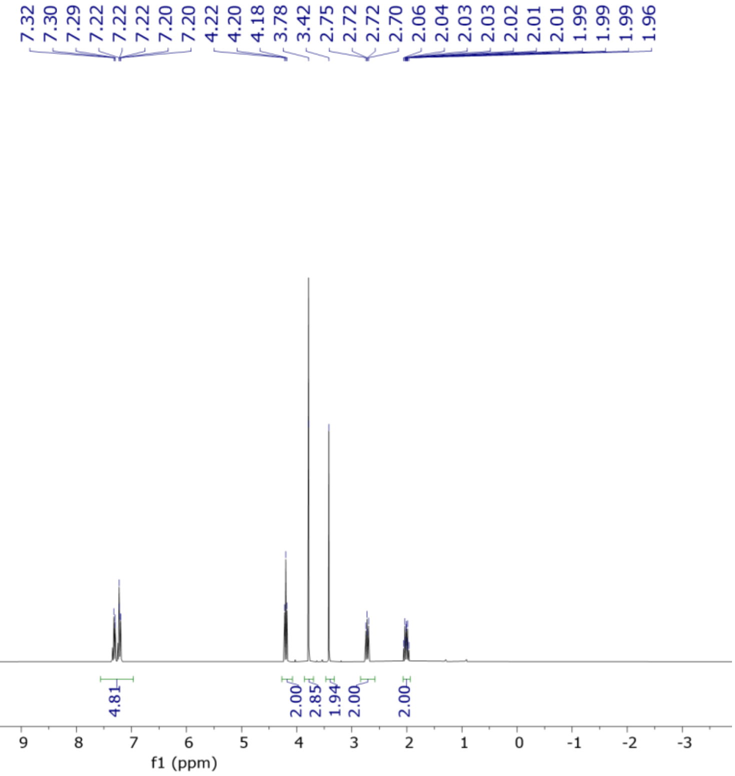

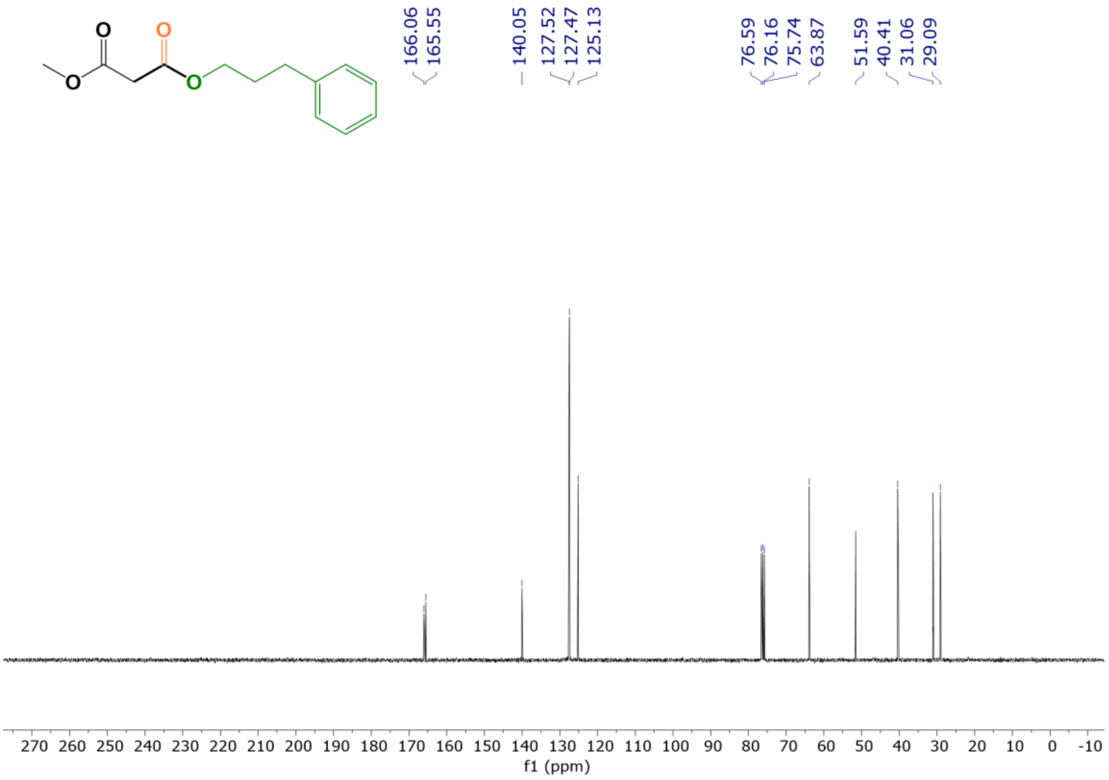

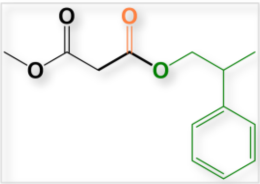

methyl (3-phenylpropyl) malonate (4). Prepared according to the general procedure. The crude product was purified by silica gel chromatography (PE/EA = 5:1) to afford the title compound as a colorless oil (505 mg, 75% yield).

^{1}H NMR (300 MHz, $CDCl_3$) δ 7.47 – 7.08 (m, 5H), 4.27 (qd, *J* = *10.8*, *7.1* Hz, 2H), 3.71 (s, 3H), 3.37 (s, 2H), 3.15 (h, *J* = *7.0* Hz, 1H), 1.33 (d, *J* = *7.0* Hz, 3H).

^{13}C NMR (75 MHz, $CDCl_3$) δ 166.85, 166.35, 142.79, 128.51, 127.26, 126.76, 52.39, 41.29, 38.78, 17.91.

HRMS (ESI): Calcd. for $C_{13}H_{16}O_4Na^+$: 259.0941, Found: 259.0942 $[M+Na]^+$.

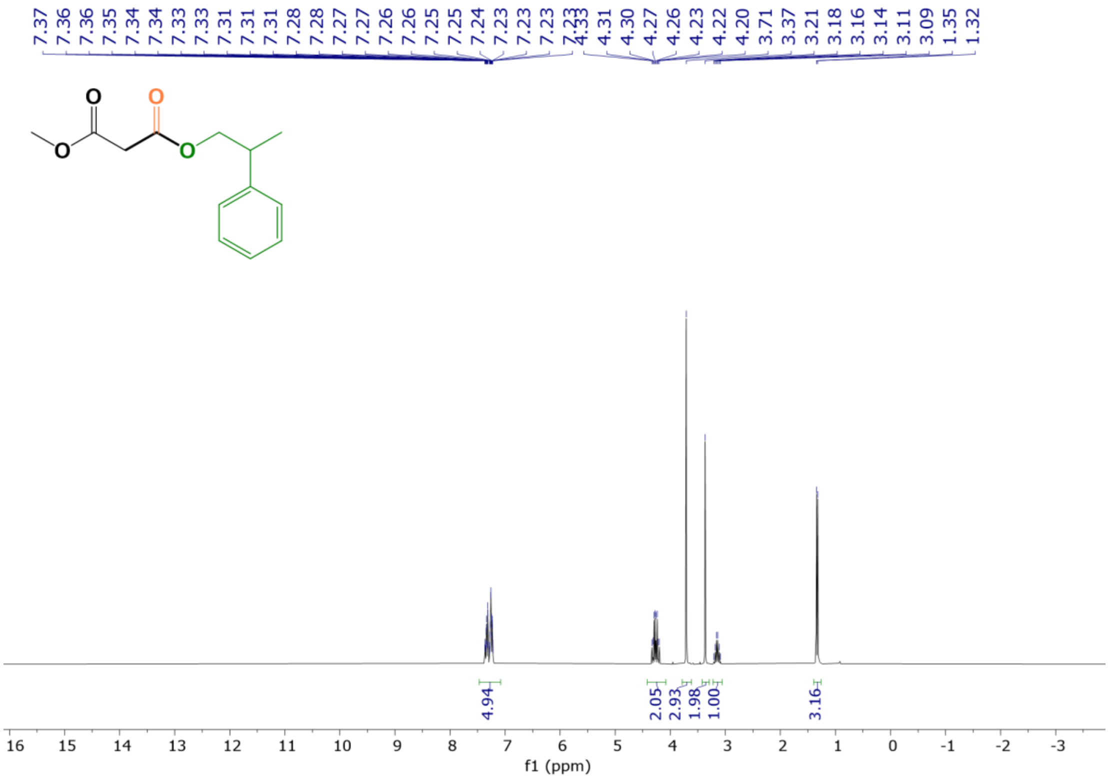
7.37 7.36 7.36 7.35 7.34 7.34 7.33 7.33 7.31 7.31 7.31 7.28 7.28 7.27 7.27 7.26 7.26 7.25 7.25 7.24 7.23 7.23 7.23 7.23 4.33 4.31 4.30 4.27 4.26 4.23 4.22 4.20 3.71 3.37 3.21 3.18 3.16 3.14 3.11 3.09 1.35 1.32
4.94
2.05
2.93
1.98
1.00
3.16
16 15 14 13 12 11 10 9 8 7 6 5 4 3 2 1 0 -1 -2 -3
f1 (ppm)

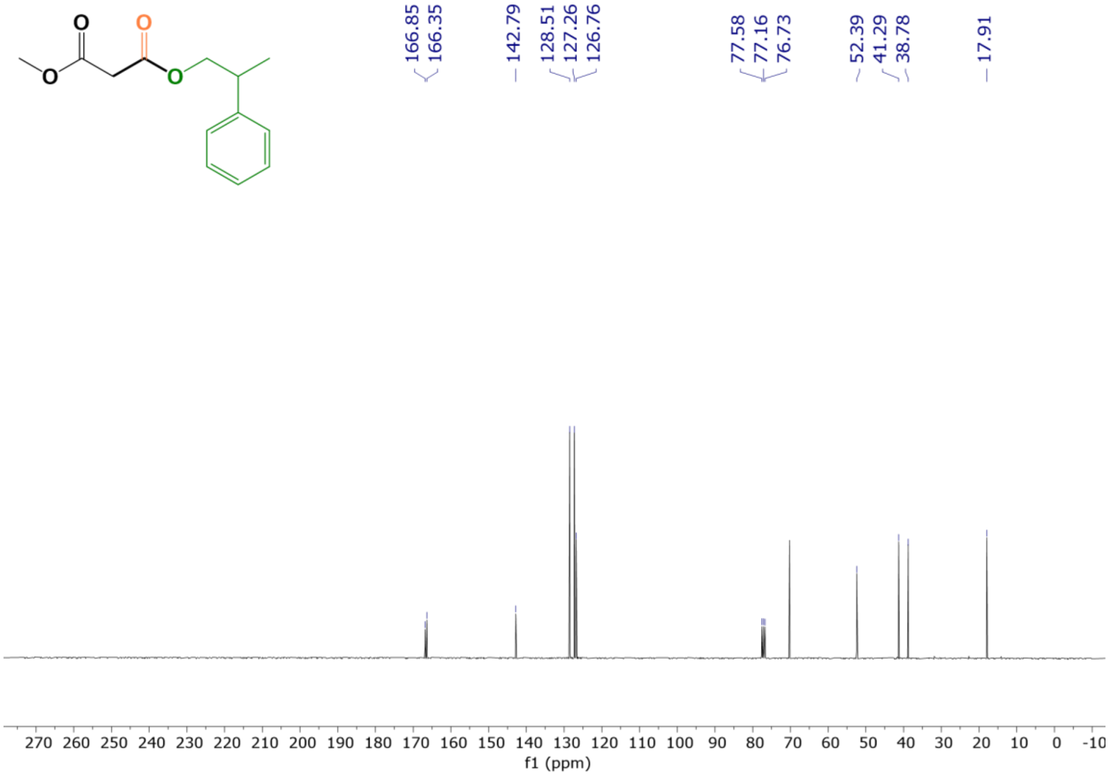
166.85 166.35
142.79
128.51 127.26 126.76
77.58 77.16 76.73
52.39 41.29 38.78
17.91
270 260 250 240 230 220 210 200 190 180 170 160 150 140 130 120 110 100 90 80 70 60 50 40 30 20 10 0 -10
f1 (ppm)

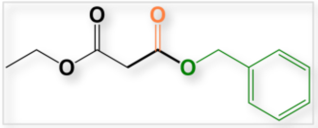

benzyl ethyl malonate (5). Known structure[4]. Prepared according to the general procedure. The crude product was purified by silica gel chromatography (PE/EA = 5:1) to afford the title compound as a colorless oil (530 mg, 85% yield).

^{1}H NMR (300 MHz, $CDCl_3$) δ 7.38 – 7.29 (m, 5H), 5.19 (s, 2H), 4.19 (q, *J* = 7.1 Hz, 2H), 3.41 (s, 2H), 1.25 (t, *J* = 7.1 Hz, 3H).

^{13}C NMR (75 MHz, $CDCl_3$) δ 166.48, 166.44, 135.37, 128.62, 128.46, 128.33, 67.19, 61.59, 41.65, 14.06.

HRMS (ESI): Calcd. for $C_{12}H_{14}O_4Na^+$: 245.0784, Found: 245.0788 $[M+Na]^+$.

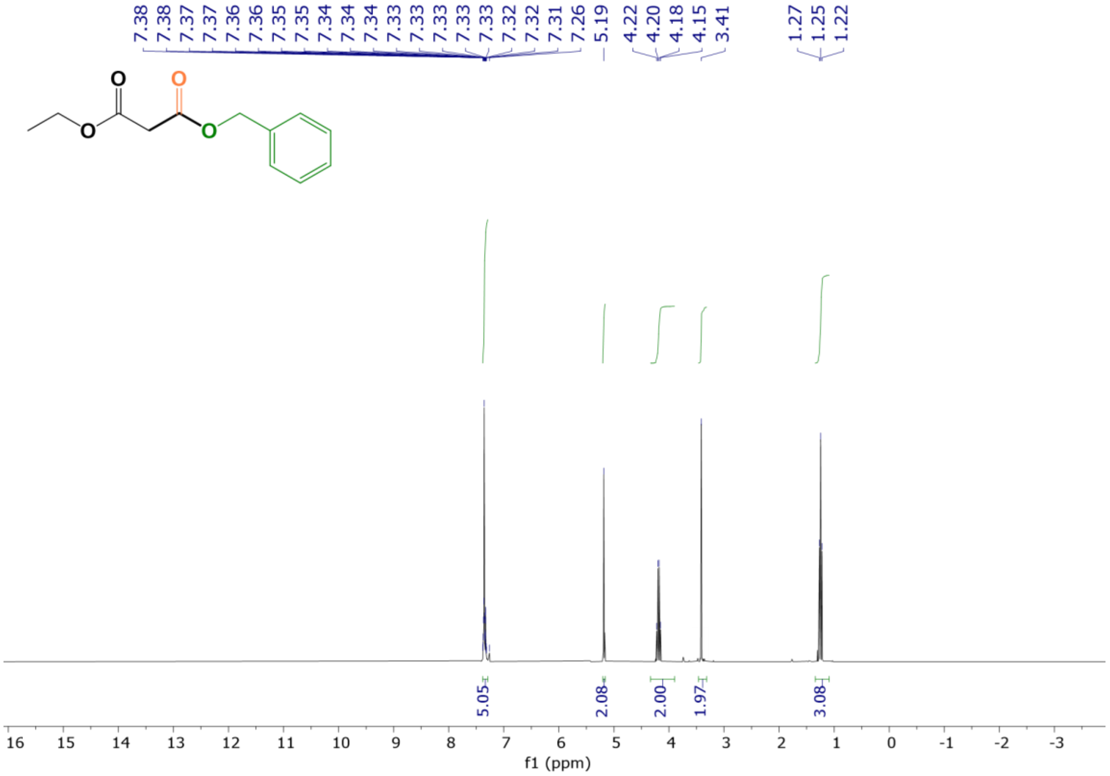

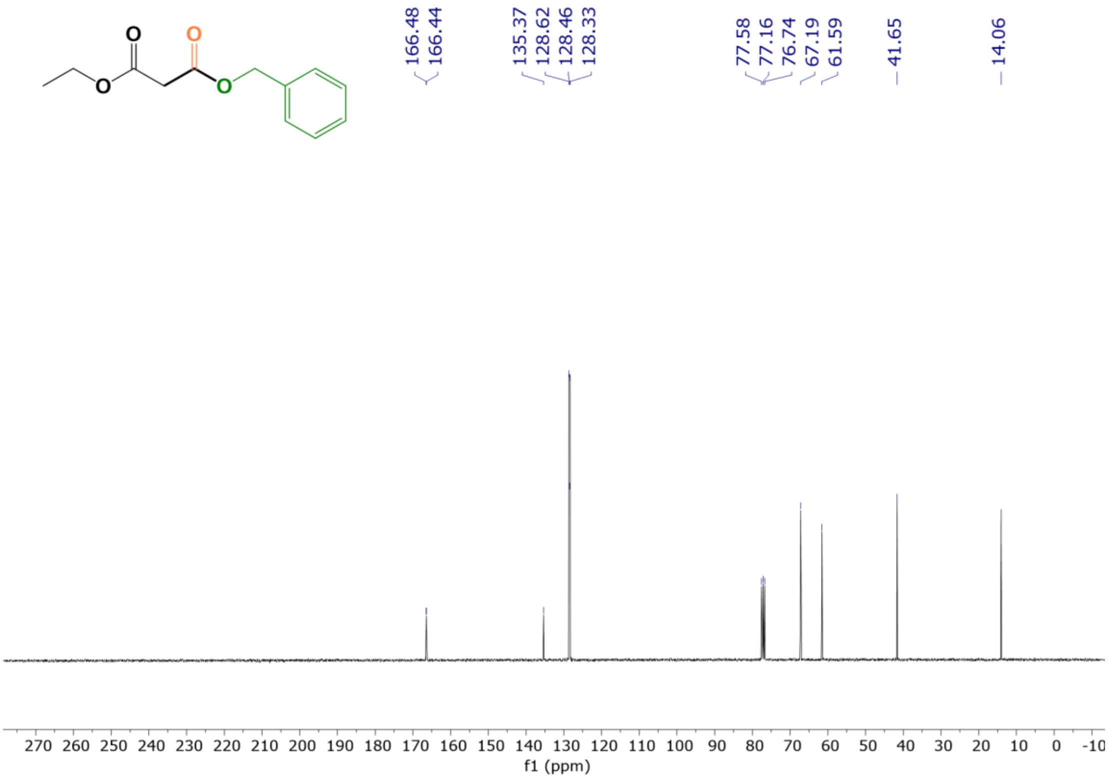

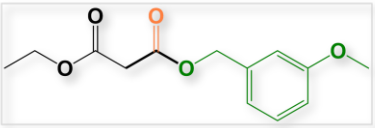

ethyl (3-methoxybenzyl) malonate (6). Prepared according to the general procedure. The crude product was purified by silica gel chromatography (PE/EA = 5:1) to afford the title compound as a colorless oil (540 mg, 75% yield).

^{1}H NMR (300 MHz, $CDCl_3$) δ 7.19 (t, *J* = 8.1 Hz, 1H), 6.97 – 6.63 (m, 3H), 5.07 (s, 2H), 4.11 (q, *J* = 7.1 Hz, 2H), 3.72 (s, 3H), 3.34 (s, 2H), 1.17 (t, *J* = 7.1 Hz, 3H).

^{13}C NMR (75 MHz, $CDCl_3$) δ 166.46, 159.81, 136.87, 129.69, 120.41, 113.98, 113.69, 67.04, 61.62, 55.28, 41.64, 14.07.

HRMS (ESI): Calcd. for $C_{13}H_{16}O_5Na^+$: 275.0890, Found: 275.0891 $[M+Na]^+$.

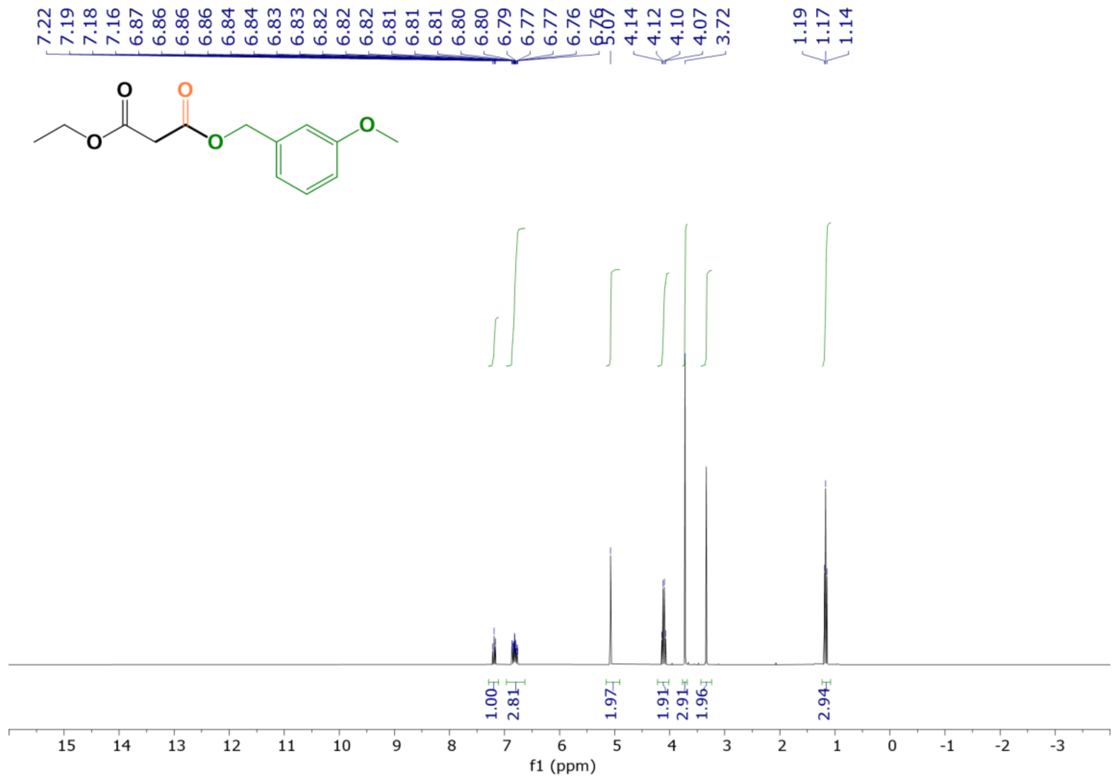
7.22 7.19 7.18 7.16 6.87 6.86 6.86 6.86 6.84 6.83 6.83 6.82 6.82 6.82 6.81 6.81 6.81 6.80 6.80 6.79 6.77 6.77 6.76 6.76 5.07 4.14 4.12 4.10 4.07 3.72 1.19 1.17 1.14
O
O
O
O
O
1.00 2.81 1.97 1.91 2.91 1.96 2.94
f1 (ppm)

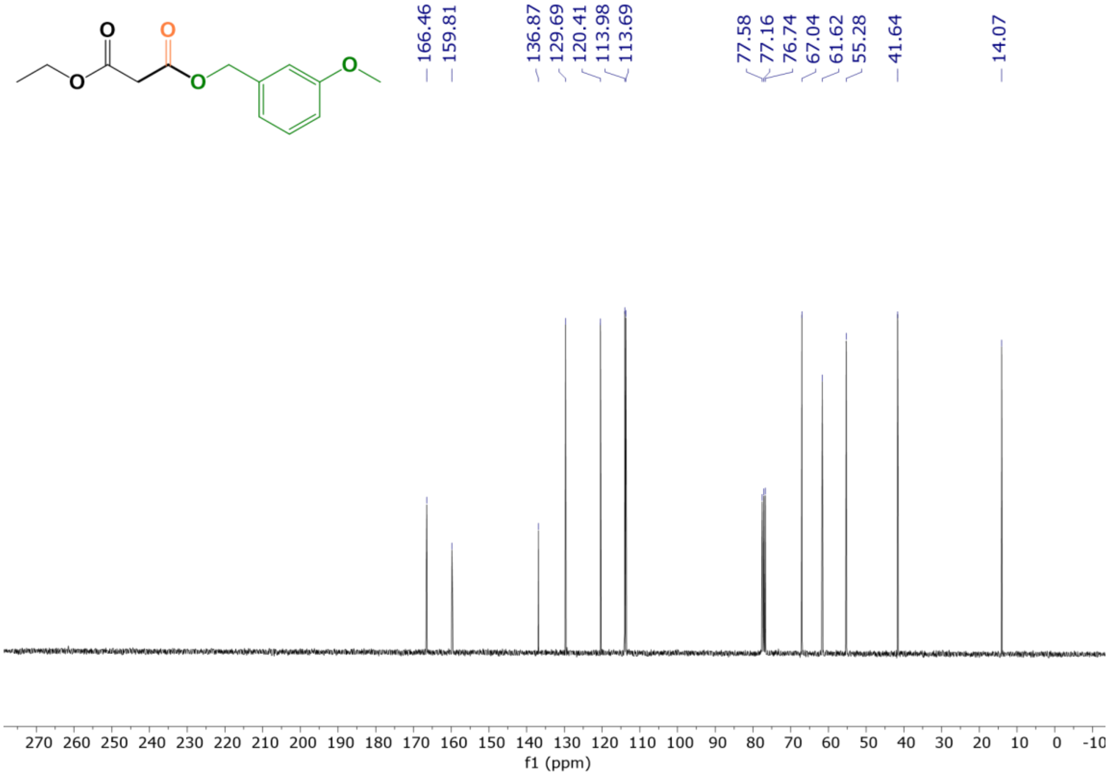
O
O
O
O
O
166.46 159.81 136.87 129.69 120.41 113.98 113.69 77.58 77.16 76.74 67.04 61.62 55.28 41.64 14.07
f1 (ppm)

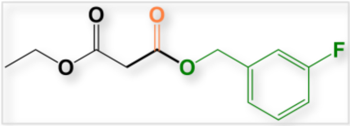

ethyl (3-fluorobenzyl) malonate (7). Prepared according to the general procedure. The crude product was purified by silica gel chromatography (PE/EA = 5:1) to afford the title compound as a colorless oil (412 mg, 60% yield).

^{1}H NMR (300 MHz, $CDCl_3$) δ 7.53 – 6.77 (m, 4H), 5.18 (s, 2H), 4.21 (q, J = 7.1 Hz, 2H), 3.44 (s, 2H), 1.26 (t, J = 7.1 Hz, 3H).

^{13}C NMR (75 MHz, $CDCl_3$) δ 166.35, 164.52, 161.25, 137.94, 137.84, 130.28, 130.17, 123.64, 123.60, 115.46, 115.18, 115.13, 114.84, 66.26, 66.23, 61.70, 41.60, 14.06.

HRMS (ESI): Calcd. for $C_{12}H_{13}FO_4Na^+$: 263.0690, Found: 263.0695 $[M+Na]^+$.

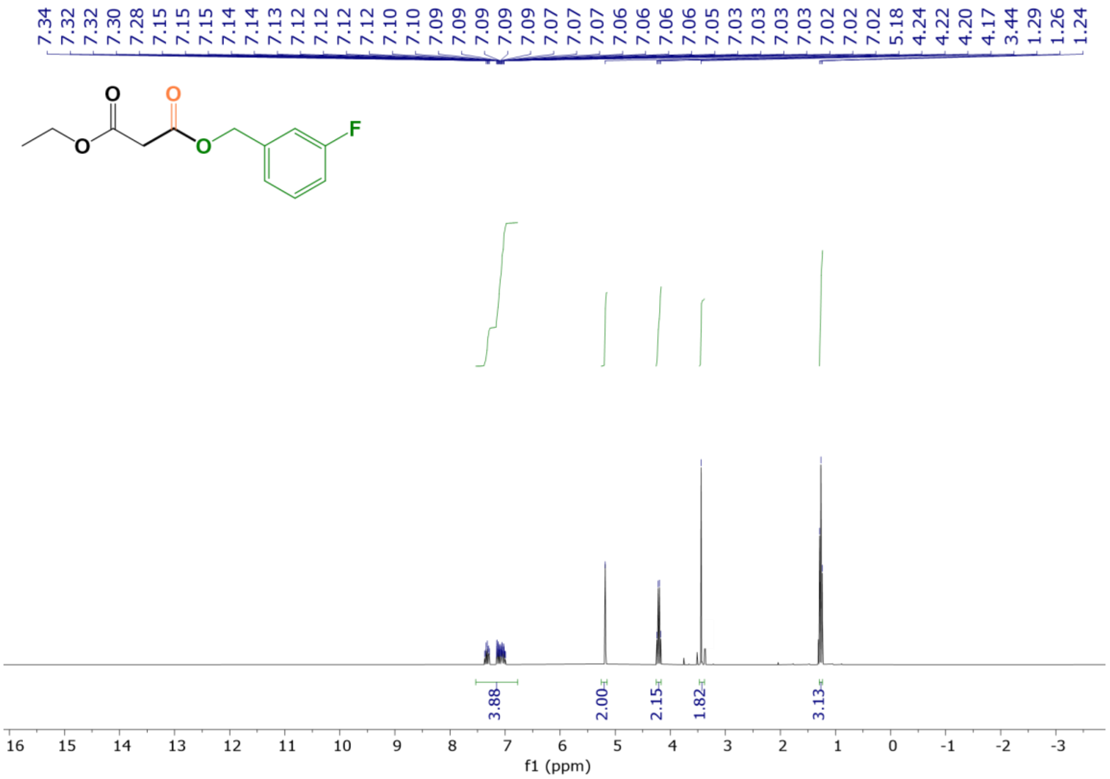

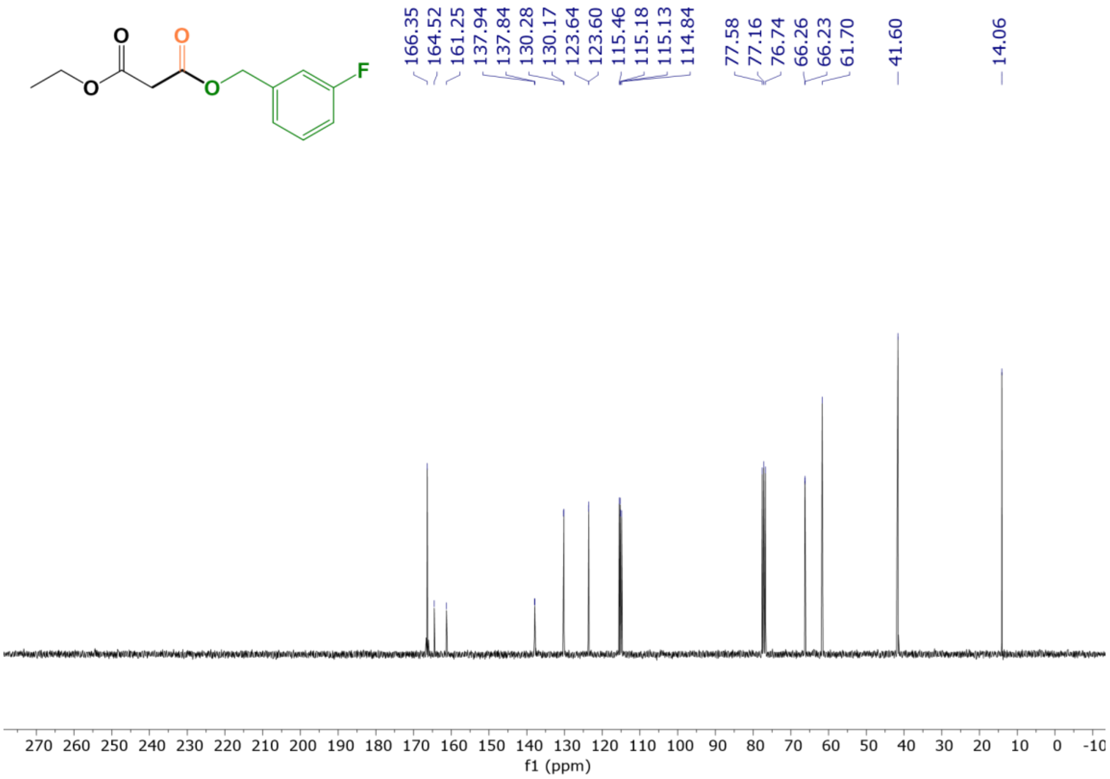

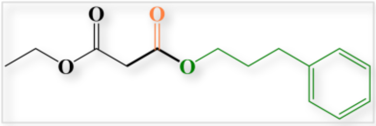

ethyl (3-phenylpropyl) malonate (8). Known structure[4]. Prepared according to the general procedure. The crude product was purified by silica gel chromatography (PE/EA = 5:1) to afford the title compound as a colorless oil (640 mg, 90% yield).

^{1}H NMR (300 MHz, $CDCl_3$) δ 7.35 – 7.18 (m, 5H), 4.40 – 4.06 (m, 4H), 3.40 (s, 2H), 2.83 – 2.61 (m, 2H), 2.13 – 1.94 (m, 2H), 1.32 (t, *J* = 7.1 Hz, 3H).

^{13}C NMR (75 MHz, $CDCl_3$) δ 166.67, 166.62, 141.05, 128.51, 128.46, 126.11, 64.79, 61.57, 41.69, 32.03, 30.10, 14.14.

HRMS (ESI): Calcd. for $C_{14}H_{18}O_4Na^+$: 273.1097, Found: 273.1105 $[M+Na]^+$.

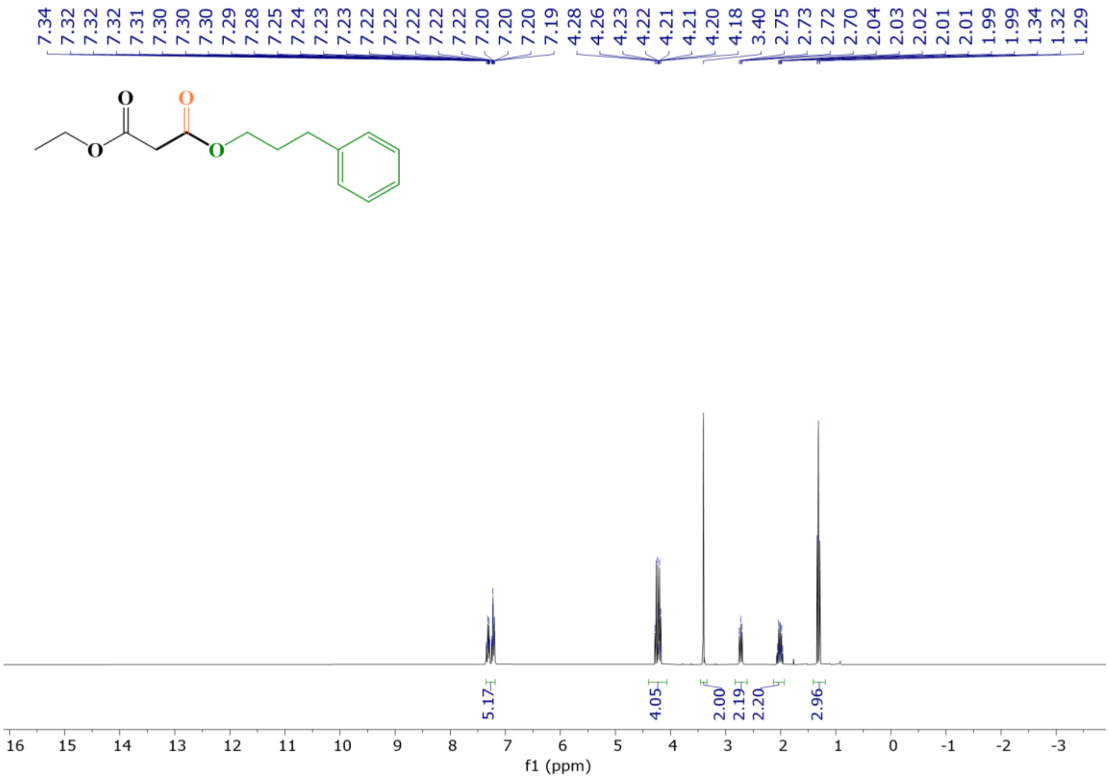
7.34
7.32
7.32
7.32
7.31
7.30
7.30
7.30
7.29
7.28
7.25
7.24
7.23
7.23
7.22
7.22
7.22
7.22
7.22
7.20
7.20
7.20
7.19
4.28
4.26
4.23
4.22
4.21
4.21
4.20
4.18
3.40
2.75
2.73
2.72
2.70
2.04
2.03
2.02
2.01
2.01
1.99
1.99
1.34
1.32
1.29
O
O
O
O
5.17
4.05
2.00
2.19
2.20
2.96
16 15 14 13 12 11 10 9 8 7 6 5 4 3 2 1 0 -1 -2 -3
f1 (ppm)

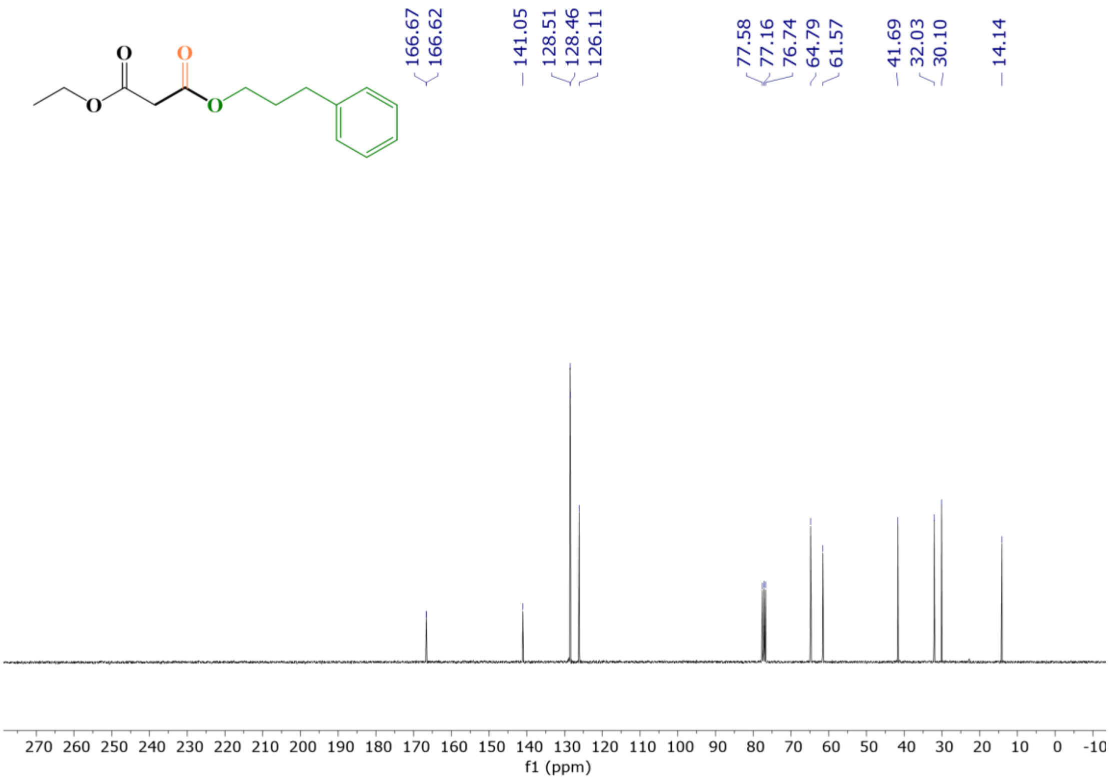
166.67
166.62
141.05
128.51
128.46
126.11
77.58
77.16
76.74
64.79
61.57
41.69
32.03
30.10
14.14
O
O
O
O
270 260 250 240 230 220 210 200 190 180 170 160 150 140 130 120 110 100 90 80 70 60 50 40 30 20 10 0 -10
f1 (ppm)

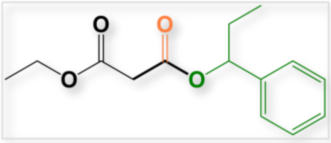

ethyl (1-phenylpropyl) malonate (9). Prepared according to the general procedure. The crude product was purified by silica gel chromatography (PE/EA = 5:1) to afford the title compound as a colorless oil (576 mg, 81% yield).

^{1}H NMR (300 MHz, CDCl$_3$) δ 7.35 – 7.08 (m, 5H), 5.64 (dd, *J* = 7.5, 6.2 Hz, 1H), 4.10 (q, *J* = 7.2 Hz, 2H), 3.31 (s, 2H), 1.98 – 1.66 (m, 2H), 1.16 (t, *J* = 7.1 Hz, 3H), 0.82 (t, *J* = 7.4 Hz, 3H).

^{13}C NMR (75 MHz, CDCl$_3$) δ 166.58, 166.00, 139.96, 128.48, 128.08, 126.63, 78.68, 61.57, 42.04, 29.30, 14.11, 9.91.

HRMS (ESI): Calcd. for $C_{14}H_{18}O_4Na^+$: 273.1097, Found: 273.1099 [M+Na]$^+$.

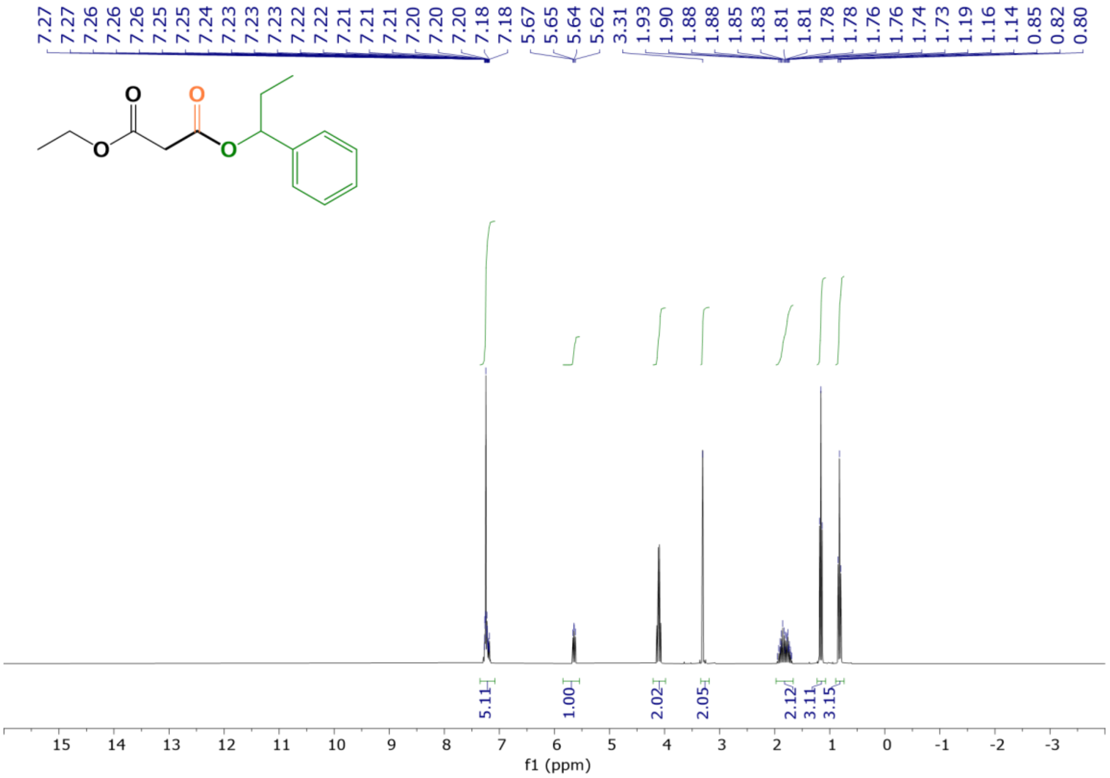

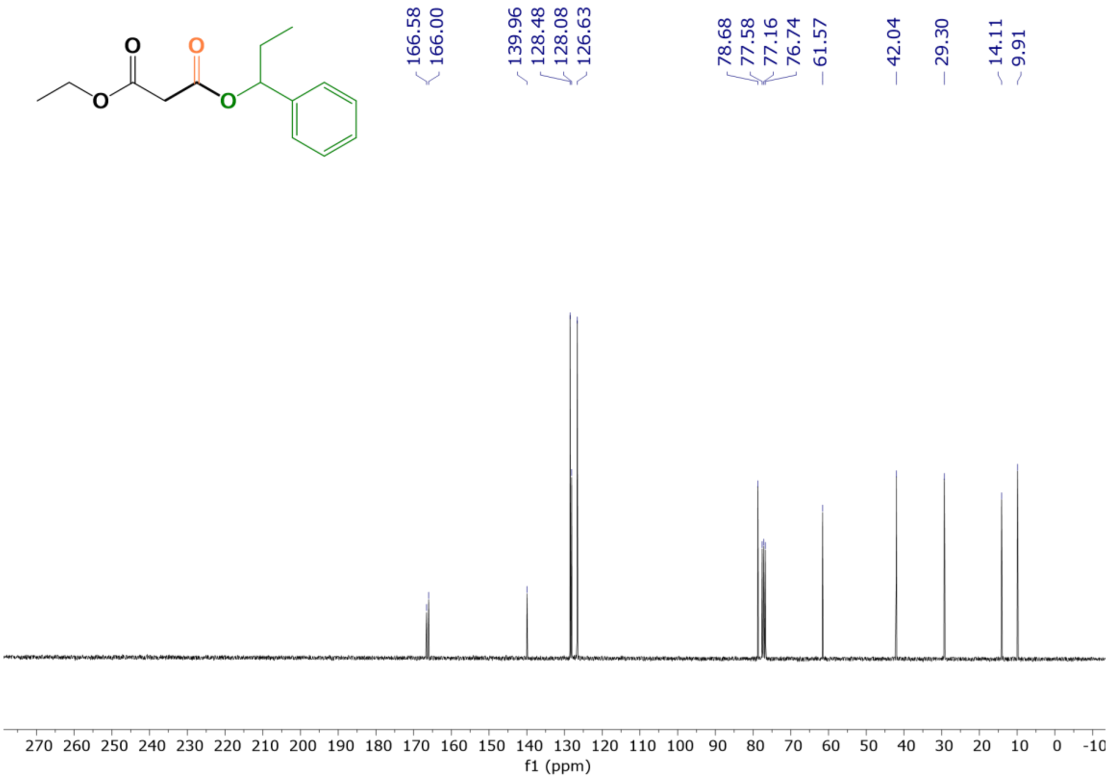

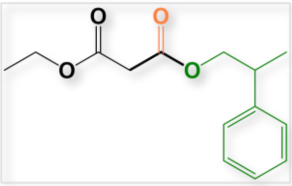

ethyl (1-phenylpropyl) malonate (10). Prepared according to the general procedure. The crude product was purified by silica gel chromatography (PE/EA = 5:1) to afford the title compound as a colorless oil (611 mg, 86% yield).

^{1}H NMR (300 MHz, CDCl$_3$) δ 7.41 – 7.18 (m, 5H), 4.54 – 4.04 (m, 4H), 3.36 (s, 2H), 3.20 – 3.07 (m, 1H), 1.41 – 1.16 (m, 6H).

^{13}C NMR (75 MHz, CDCl$_3$) δ 166.48, 166.42, 142.80, 128.51, 127.25, 126.75, 70.22, 61.45, 41.57, 38.79, 17.93, 14.02.

HRMS (ESI): Calcd. for $C_{14}H_{18}O_4Na^+$: 273.1097, Found: 273.1098 [M+Na]$^+$.

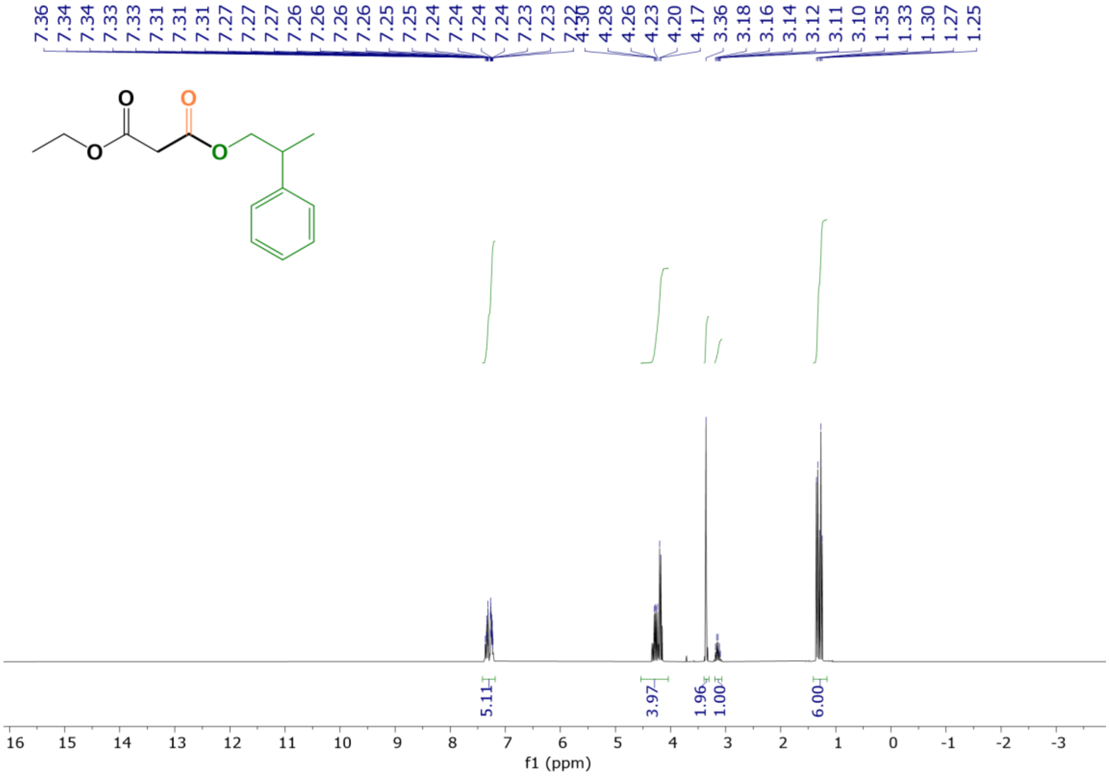
7.36 7.34 7.34 7.33 7.33 7.31 7.31 7.31 7.27 7.27 7.27 7.26 7.26 7.26 7.26 7.25 7.25 7.24 7.24 7.24 7.24 7.23 7.23 7.22 4.30 4.28 4.26 4.23 4.20 4.17 3.36 3.18 3.16 3.14 3.12 3.11 3.10 1.35 1.33 1.30 1.27 1.25
5.11
3.97
1.96
1.00
6.00
16 15 14 13 12 11 10 9 8 7 6 5 4 3 2 1 0 -1 -2 -3
f1 (ppm)

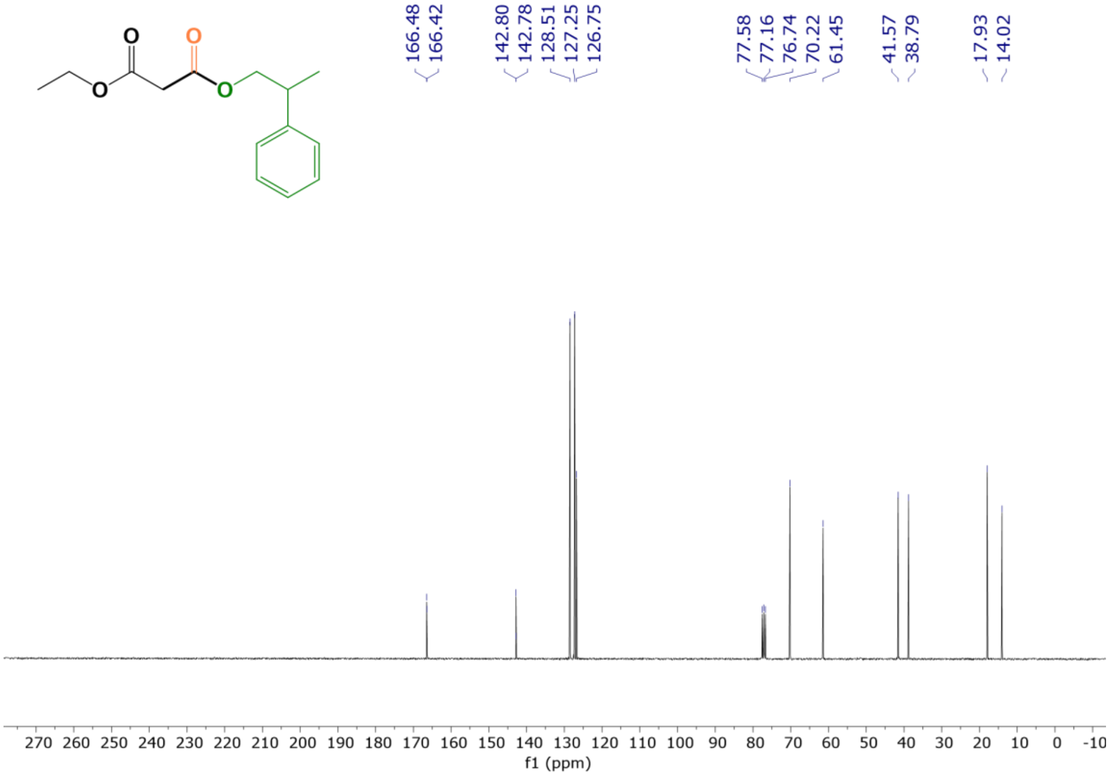
166.48 166.42
142.80 142.78 128.51 127.25 126.75
77.58 77.16 76.74 70.22 61.45
41.57 38.79
17.93 14.02
270 260 250 240 230 220 210 200 190 180 170 160 150 140 130 120 110 100 90 80 70 60 50 40 30 20 10 0 -10
f1 (ppm)

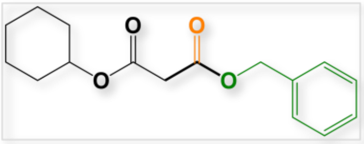

benzyl cyclohexyl malonate (11). Prepared according to the general procedure. The crude product was purified by silica gel chromatography (PE/EA = 5:1) to afford the title compound as a colorless oil (583 mg, 75% yield).

^{1}H NMR (300 MHz, CDCl$_3$) δ 7.40 – 7.29 (m, 5H), 5.18 (s, 2H), 4.80 (td, *J* = 8.8, 3.9 Hz, 1H), 3.40 (s, 2H), 1.84 – 1.21 (m, 10H).

^{13}C NMR (75 MHz, CDCl$_3$) δ 166.65, 165.95, 135.44, 128.66, 128.49, 128.44, 67.21, 42.09, 31.41, 25.37, 23.64.

HRMS (ESI): Calcd. for $C_{16}H_{20}O_4Na^+$: 299.1254, Found: 299.1255 $[M+Na]^+$.

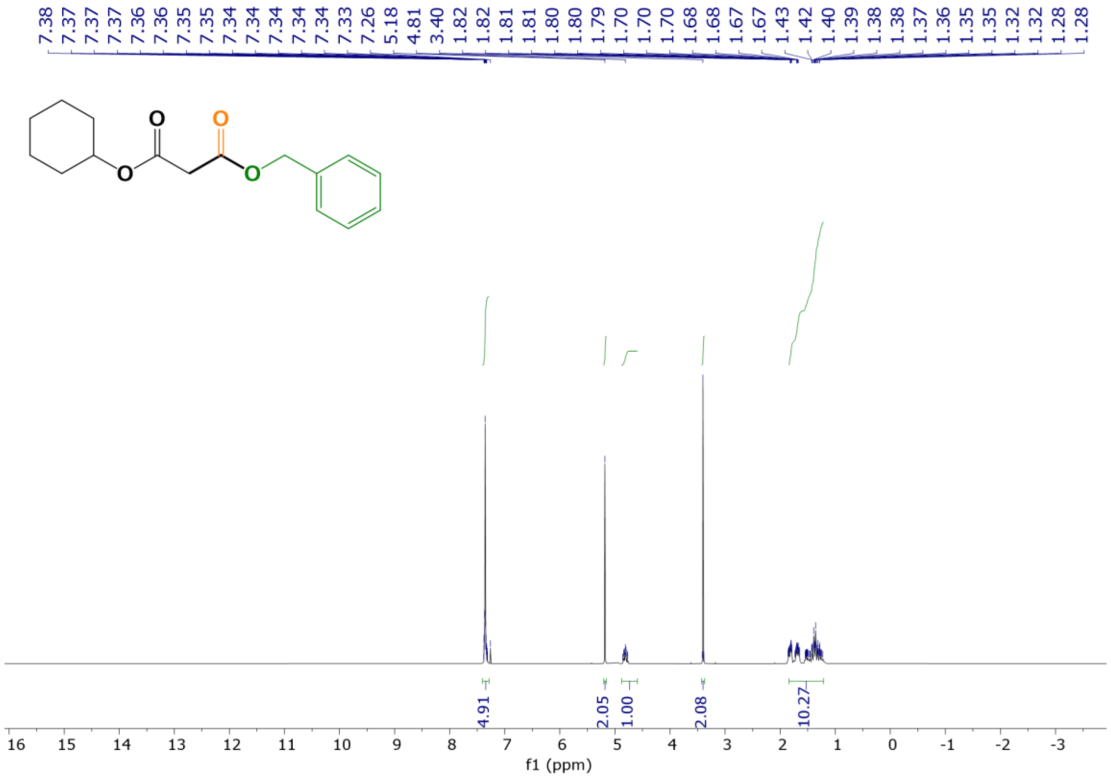

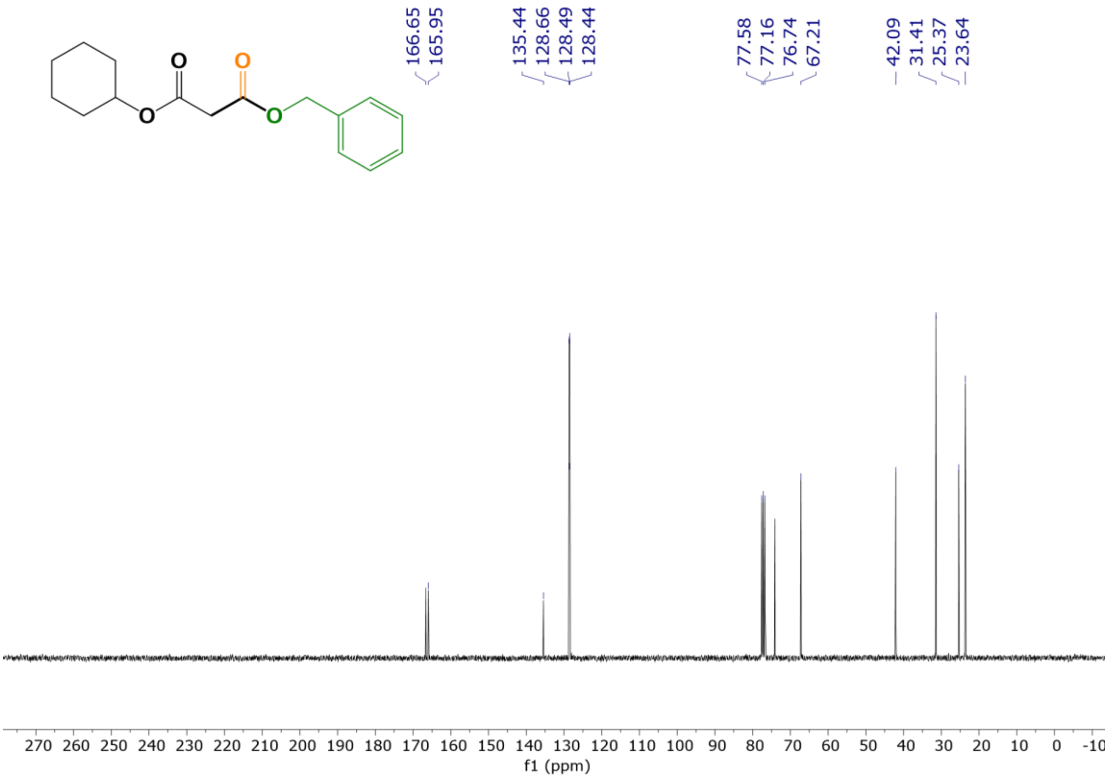

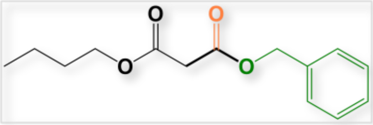

benzyl butyl malonate (12). Prepared according to the general procedure. The crude product was purified by silica gel chromatography (PE/EA = 5:1) to afford the title compound as a little yellow oil (584 mg, 82% yield).

^{1}H NMR (400 MHz, $CDCl_3$) δ 7.39 – 7.30 (m, 5H), 5.18 (s, 2H), 4.14 (t, *J* = 6.7 Hz, 2H), 3.42 (s, 2H), 1.59 (ddt, *J* = 8.8, 7.9, 6.6 Hz, 2H), 1.40 – 1.30 (m, 2H), 0.91 (t, *J* = 7.4 Hz, 3H).

^{13}C NMR (101 MHz, $CDCl_3$) δ 166.56, 166.51, 135.37, 128.64, 128.49, 128.38, 67.24, 65.50, 41.69, 30.50, 19.05, 13.71.

HRMS (ESI): Calcd. for $C_{14}H_{18}O_4Na^+$: 273.1097, Found: 273.1102 $[M+Na]^+$.

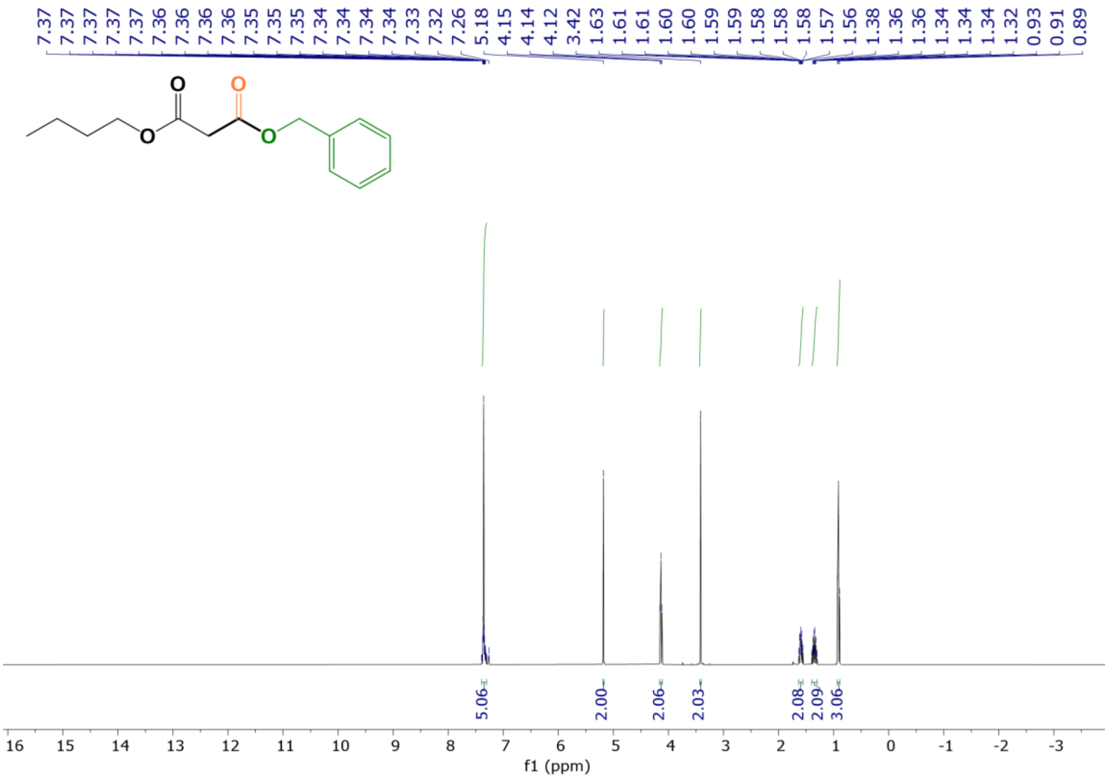

7.37
7.37
7.37
7.37
7.37
7.36
7.36
7.36
7.36
7.35
7.35
7.35
7.34
7.34
7.34
7.34
7.33
7.32
7.26
5.18
4.15
4.14
4.12
3.42
1.63
1.61
1.61
1.60
1.60
1.59
1.59
1.58
1.58
1.58
1.57
1.56
1.38
1.36
1.36
1.34
1.34
1.34
1.32
0.93
0.91
0.89
5.06
2.00
2.06
2.03
2.08
2.09
3.06
f1 (ppm)

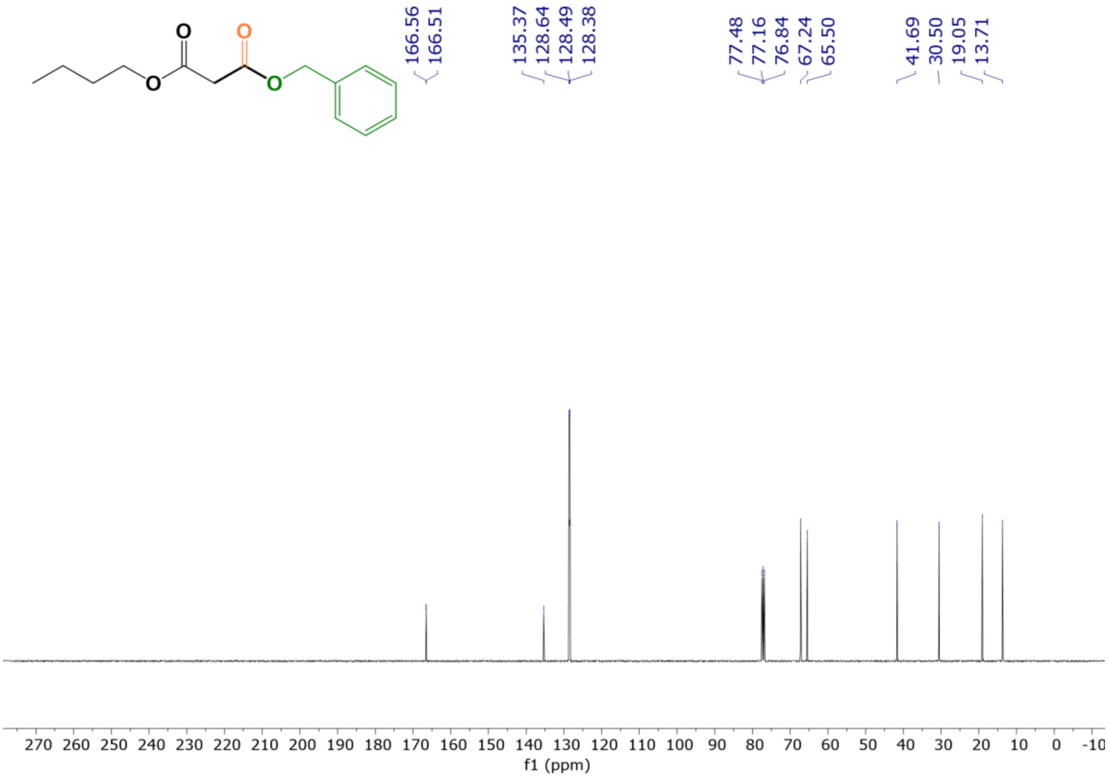

166.56
166.51
135.37
128.64
128.49
128.38
77.48
77.16
76.84
67.24
65.50
41.69
30.50
19.05
13.71
f1 (ppm)

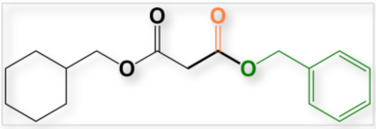

benzyl (cyclohexylmethyl) malonate (13). Prepared according to the general procedure. The crude product was purified by silica gel chromatography (PE/EA = 5:1) to afford the title compound as a little yellow oil (595 mg, 72% yield).

^{1}H NMR (300 MHz, $CDCl_3$) δ 7.43 – 7.28 (m, 5H), 5.18 (s, 2H), 3.95 (d, J = 6.5 Hz, 2H), 3.43 (s, 2H), 1.74 – 1.61 (m, 6H), 1.28 – 0.89 (m, 5H).

^{13}C NMR (75 MHz, $CDCl_3$) δ 166.62, 166.57, 135.39, 128.70, 128.54, 128.43, 70.79, 67.32, 41.74, 37.02, 29.60, 26.39, 25.71.

HRMS (ESI): Calcd. for $C_{17}H_{22}O_4Na^+$: 313.1410, Found: 313.1413 $[M+Na]^+$.

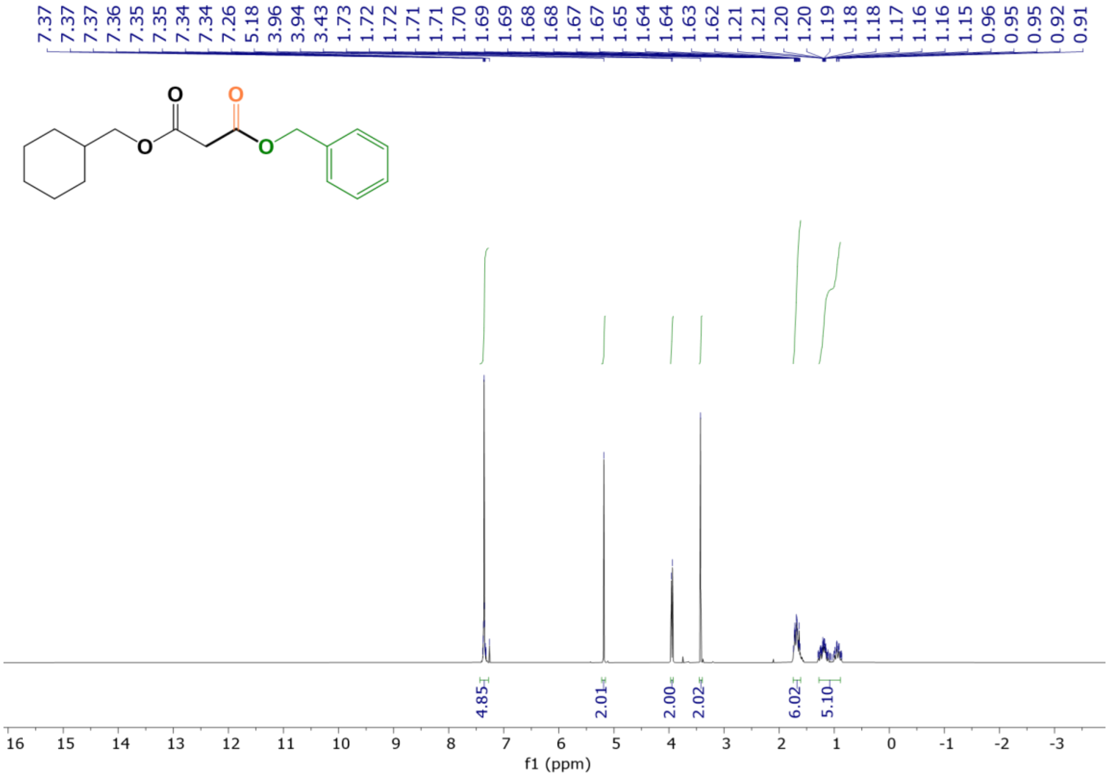

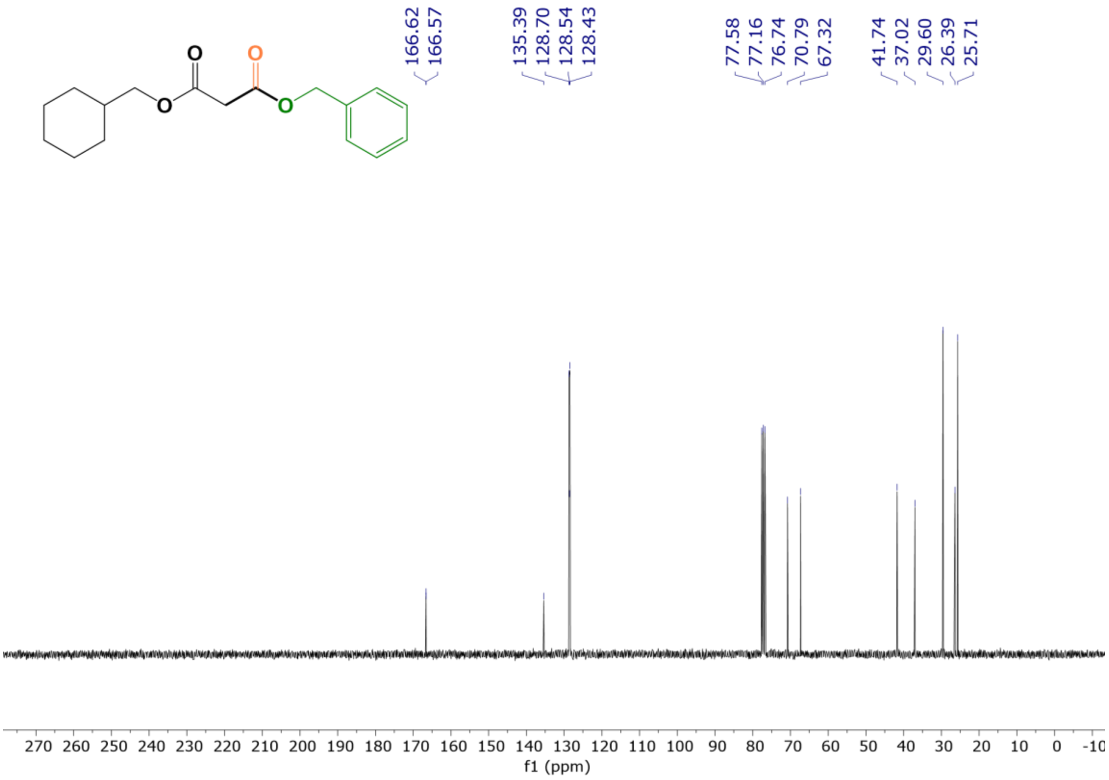

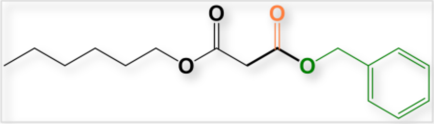

benzyl hexyl malonate (14). Prepared according to the general procedure. The crude product was purified by silica gel chromatography (PE/EA = 5:1) to afford the title compound as a colorless oil (617 mg, 78% yield).

^{1}H NMR (300 MHz, $CDCl_3$) δ 7.41 – 7.28 (m, 5H), 5.18 (s, 2H), 4.13 (t, J = 6.8 Hz, 2H), 3.42 (s, 2H), 1.66 – 1.55 (m, 2H), 1.36 – 1.24 (m, 6H), 0.92 – 0.85 (m, 3H).

^{13}C NMR (75 MHz, $CDCl_3$) δ 166.56, 166.51, 135.39, 128.65, 128.48, 128.35, 67.23, 65.81, 41.69, 31.42, 28.45, 25.49, 22.56, 14.05.

HRMS (ESI): Calcd. for $C_{16}H_{22}O_4Na^+$: 301.1410, Found: 301.1411 $[M+Na]^+$.

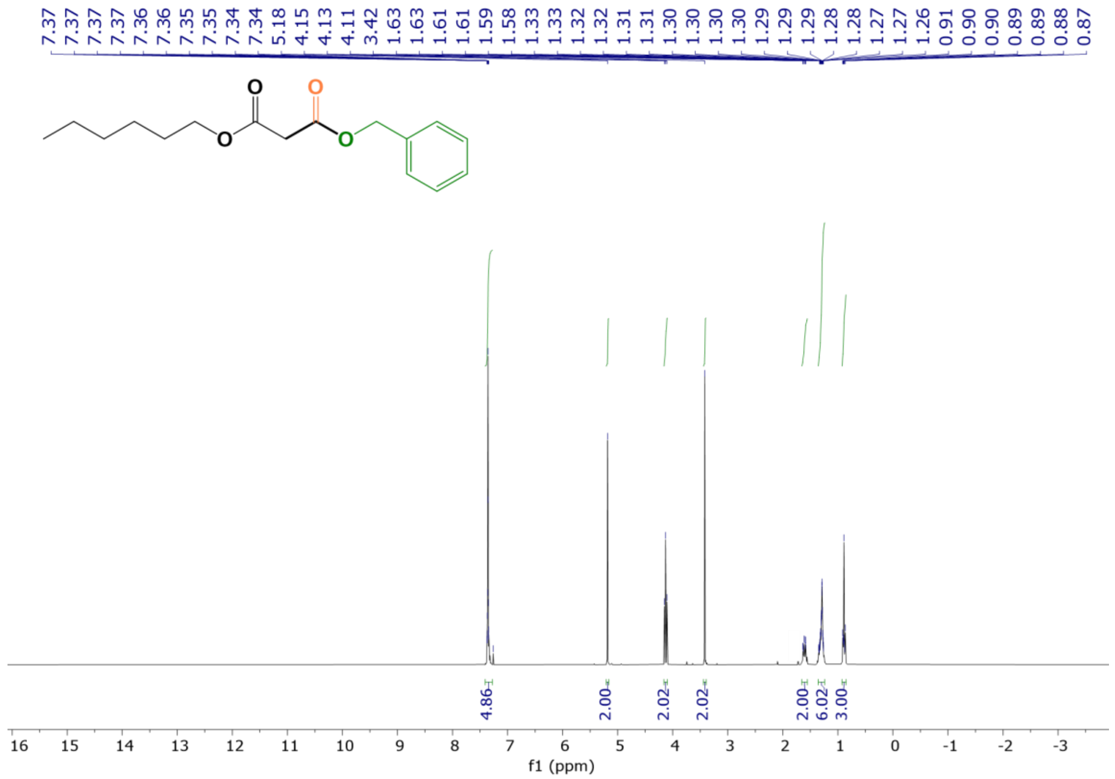
7.37
7.37
7.37
7.37
7.36
7.36
7.35
7.35
7.34
7.34
5.18
4.15
4.13
4.11
3.42
1.63
1.63
1.61
1.61
1.59
1.58
1.33
1.33
1.32
1.32
1.31
1.31
1.30
1.30
1.30
1.30
1.29
1.29
1.29
1.28
1.27
1.27
1.26
0.91
0.90
0.90
0.89
0.89
0.88
0.87
4.86
2.00
2.02
2.02
2.00
6.02
3.00
f1 (ppm)

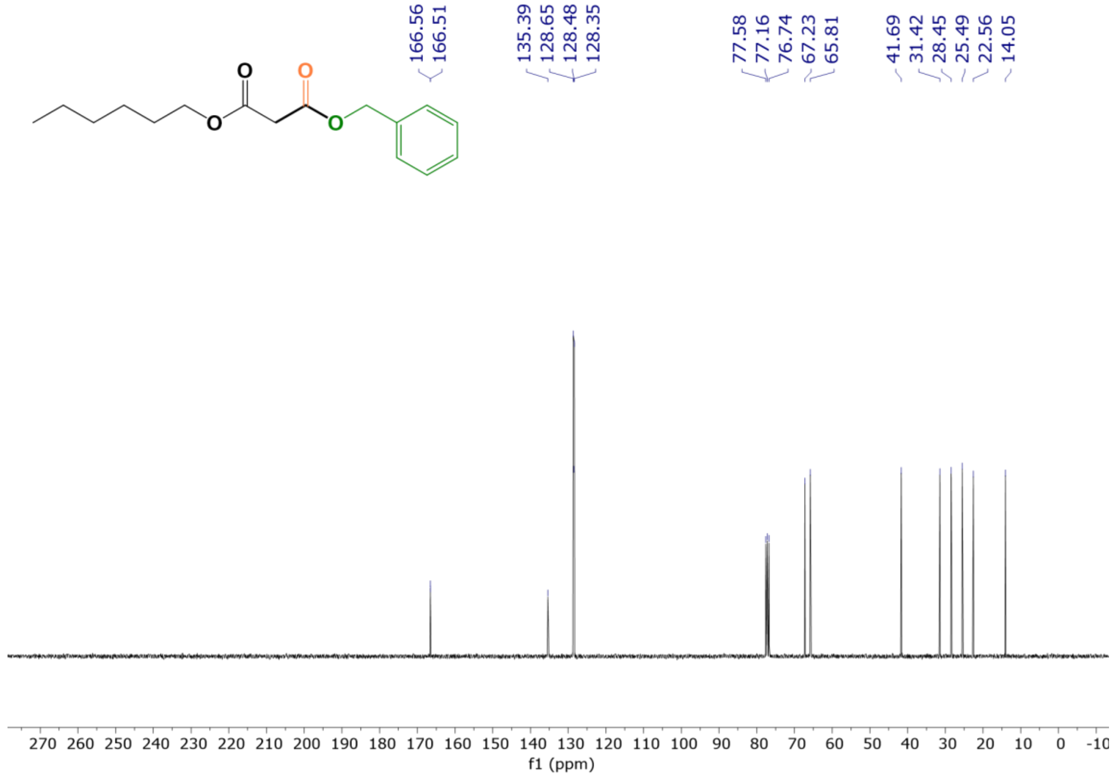
166.56
166.51
135.39
128.65
128.48
128.35
77.58
77.16
76.74
67.23
65.81
41.69
31.42
28.45
25.49
22.56
14.05
f1 (ppm)

7. Reference

[1] D. P. Curran, C. P. Jasperse, M. J. Totleben, *J. Org. Chem.* **1991**, *56*, 7169-7172.

[2] F. Rajabi, C. Wilhelm, W. R. Thiel, *Green Chem.* **2020**, *22*, 4438-4444.

[3] H. Ledon, G. Linstrumelle, S. Julia, *Tetrahedron* **1973**, *29*, 3609-3617.

[4] H. Yang, Z.-P. Bao, L.-C. Wang, X.-F. Wu, *Org. Lett.* **2023**, *25*, 1963-1968.
